# Supplementary material for: Development, maturation, and maintenance of human prostate inferred from somatic mutations
Source: Cell Stem Cell. 2021 Jul 1;28(7):1262–1274.e5. doi: 10.1016/j.stem.2021.02.005 (PMC8260206; doi:10.1016/j.stem.2021.02.005)
Supplement: Document S1. Figures S1–S6 and Tables S1 and S2 [file mmc1.pdf]

Cell Stem Cell, Volume *28*

Supplemental Information

Development, maturation, and maintenance of human prostate inferred from somatic mutations

Sebastian Grossmann, Yvette Hooks, Laura Wilson, Luiza Moore, Laura O'Neill, Iñigo Martincorena, Thierry Voet, Michael R. Stratton, Rakesh Heer, and Peter J. Campbell

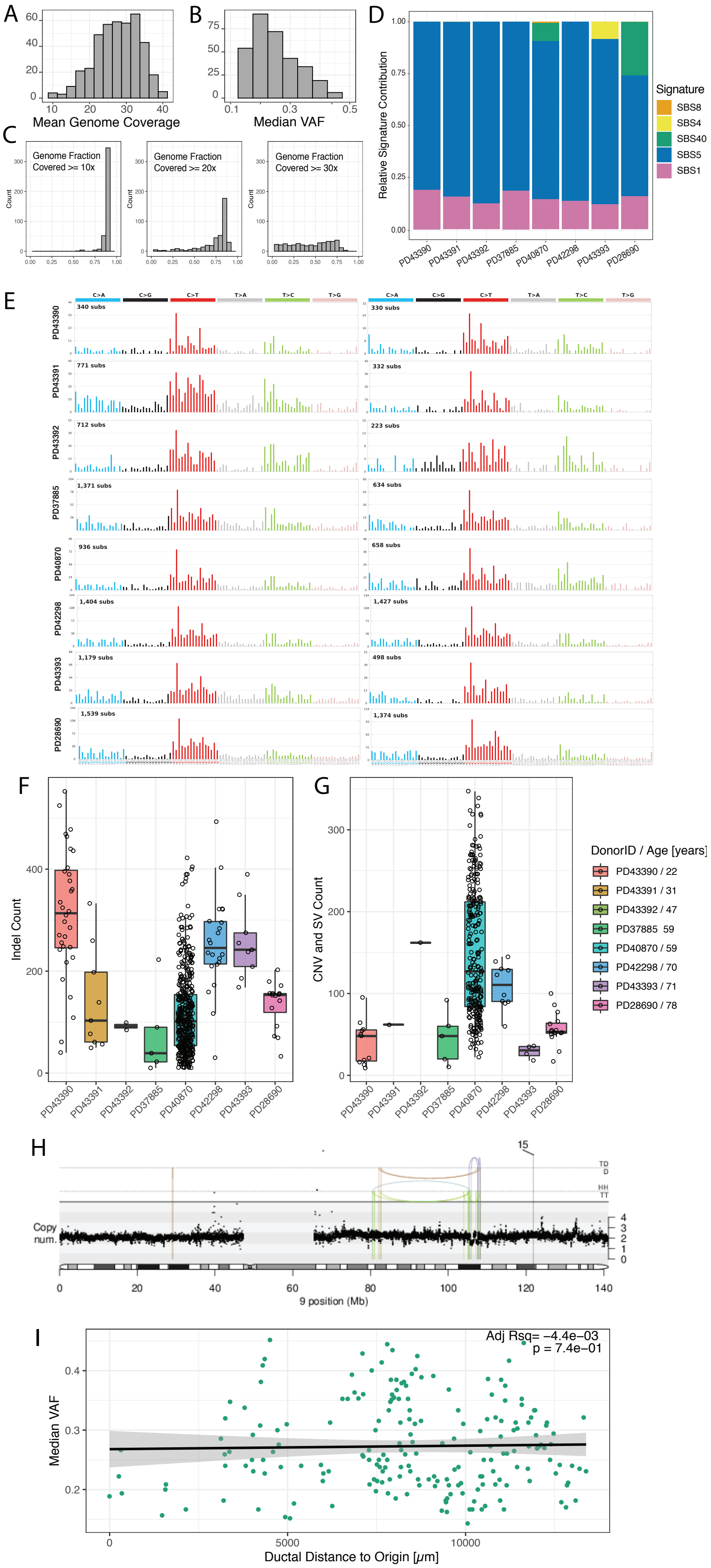
A
Mean Genome Coverage
B
Median VAF
C
Genome Fraction Covered >= 10x
Genome Fraction Covered >= 20x
Genome Fraction Covered >= 30x
Count
D
Relative Signature Contribution
Signature
SBS8
SBS4
SBS40
SBS5
SBS1
PD43390
PD43391
PD43392
PD37885
PD40870
PD42298
PD43393
PD28690
E
C>A
C>G
C>T
T>A
T>C
T>G
340 subs
330 subs
771 subs
332 subs
712 subs
223 subs
1,371 subs
634 subs
936 subs
658 subs
1,404 subs
1,427 subs
1,179 subs
498 subs
1,539 subs
1,374 subs
F
Indel Count
G
CNV and SV Count
DonorID / Age [years]
PD43390 / 22
PD43391 / 31
PD43392 / 47
PD37885 59
PD40870 / 59
PD42298 / 70
PD43393 / 71
PD28690 / 78
H
15
TD
D
HH
TT
Copy num.
9 position (Mb)
I
Adj Rsq= −4.4e−03
p = 7.4e−01
Median VAF
Ductal Distance to Origin [μm]

Supplementary Figure 1 (related to Figure 1): Genome Sequencing Quality Metrics and Mutational Landscape of Normal Prostate Epithelium. (A) Mean genome coverage per sample. **(B)** Median variant allele fraction (VAF) of single base substitutions as a measure of clonality per microdissection. **(C)** Genome Fraction per sample with coverage of at least 10x/20x/30x. **(D)** Relative contribution of deconvoluted mutational signatures per donor. The clock-like mutational signatures SBS1 and SBS5 dominate the signature landscape across all donors. **(E)** Exemplary spectral signatures of base substitutions per donor. **(F)** Count of small insertions and deletions per sample. **(G)** Only few, short copy number variants and very rare structural variants were detected in normal prostatic epithelium. **(H)** One histologically normal microdissection of prostatic epithelium contained a case of chromothripsis on chromosome 9-q. **(I)** Median VAF of base substitutions per microdissection is not correlated to the position within the ductal network.

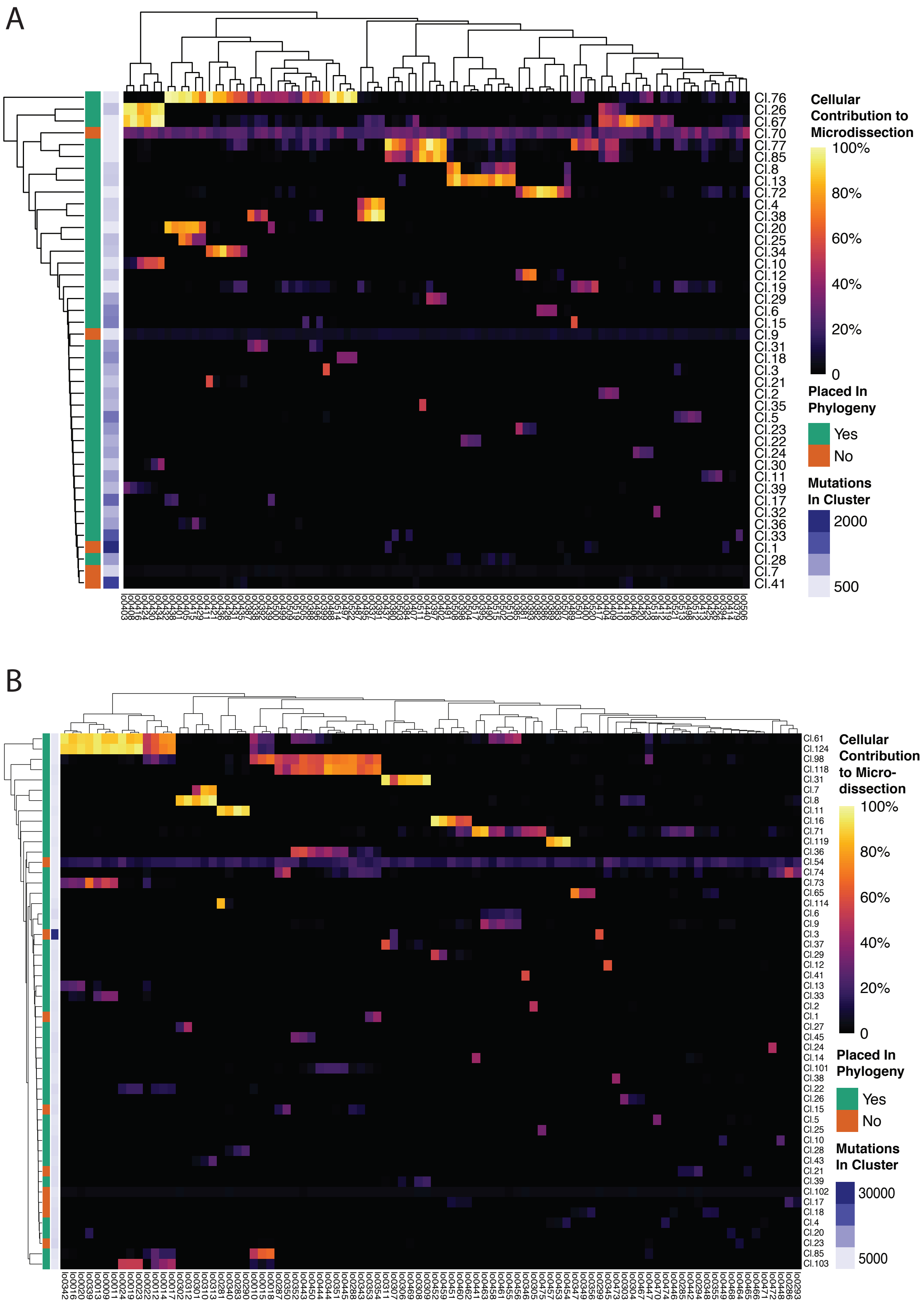
A
Cl.76
Cl.26
Cl.67
Cl.70
Cl.77
Cl.85
Cl.8
Cl.13
Cl.72
Cl.4
Cl.38
Cl.20
Cl.25
Cl.34
Cl.10
Cl.12
Cl.19
Cl.29
Cl.6
Cl.15
Cl.9
Cl.31
Cl.18
Cl.3
Cl.21
Cl.2
Cl.35
Cl.5
Cl.23
Cl.22
Cl.24
Cl.30
Cl.11
Cl.39
Cl.17
Cl.32
Cl.36
Cl.33
Cl.1
Cl.28
Cl.7
Cl.41
Cellular Contribution to Microdissection
100%
80%
60%
40%
20%
0
Placed In Phylogeny
Yes
No
Mutations In Cluster
2000
500
B
Cellular Contribution to Micro-dissection
100%
80%
60%
40%
20%
0
Placed In Phylogeny
Yes
No
Mutations In Cluster
30000
5000

Supplementary Figure 2 (related to Figure 2): Discrete Clusters of Mutations Identify Ancestral Clones that Contribute to Current Prostatic Epithelium. Clusters of mutations are identified based on VAF using an n-dimensional Hierarchical Dirichlet Process (n-HDP) clustering algorithm and constitute the rows of the heatmap. These clusters of mutations represent ancestral clones which contribute to current cells sampled in individual microdissections, which are shown in the columns of the heatmap. The median VAF of cells associated with an ancestral cluster is used to estimate the cellular contribution to each microdissection and is visualized using color intensities. The co-occurrence of multiple ancestral clones within the same microdissection sample and their corresponding cellular contribution was used to construct the lineage tree for individual glandular subunits. **(A) Heatmap for the left-hand side structure.** Cluster 70, 9 and 7 include nearly exclusively artefactual mutations and were excluded from the lineage tree. Furthermore, cluster 1 and 41 are insufficiently split by the clustering algorithm used and could not be placed unambiguously. **(B) Heatmap for the right-hand side structure.** Cluster 54 and 102 contain artefactual mutations. Seven additional clusters could not be placed onto the lineage tree unambiguously.

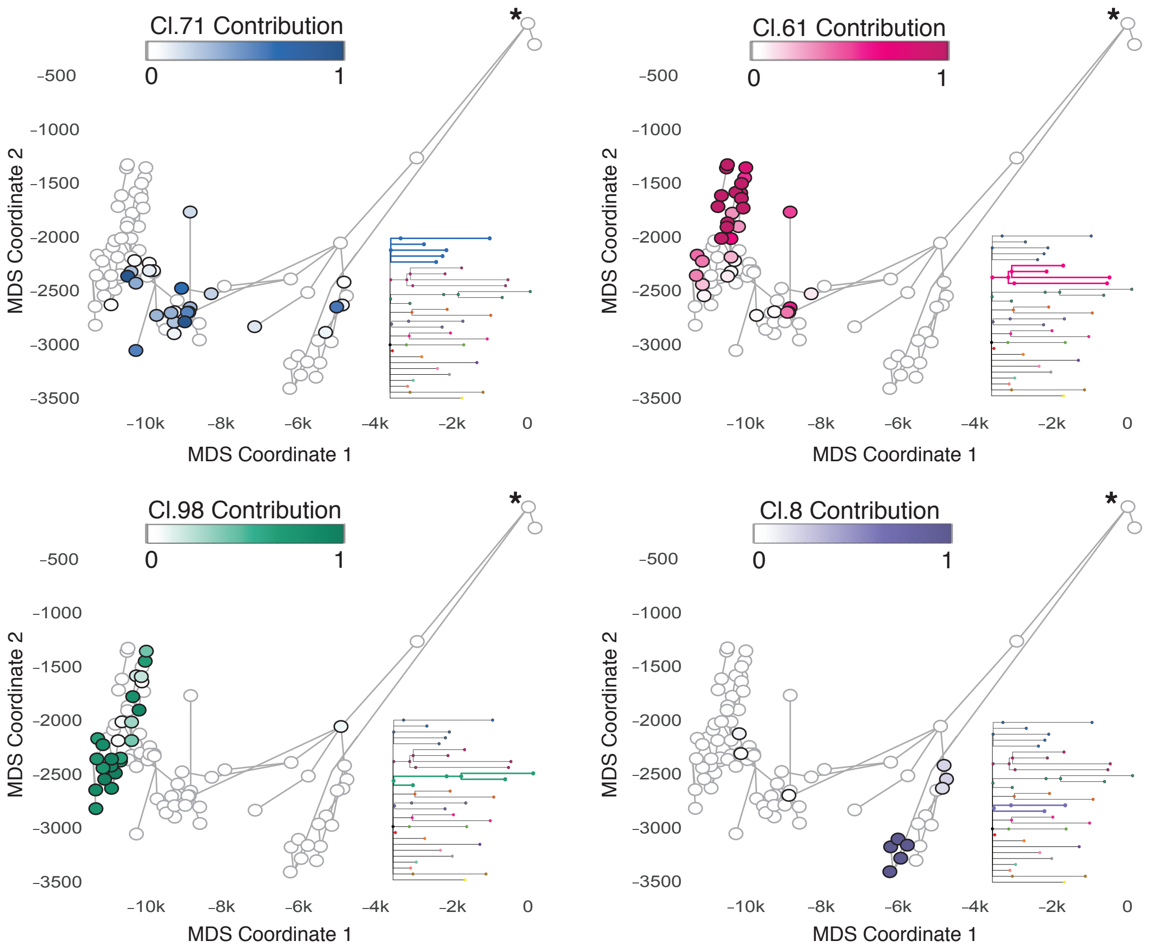

Supplementary Figure 3 (related to Figure 3): Cellular Contribution of Embryonic Clones for Glandular Subunit on the Right-Hand Side. The cellular contribution of four ancestral clones from embryonic development is displayed for the glandular subunit from the right-hand side. Each circle marks a microdissection and the ductal connection is indicated by grey lines. Microdissections with positive contribution from the clone are circled in black, while those with zero contribution are circled in grey. The most urethra-proximal microdissection is marked with an asterisk. Three embryonic clones display a wide and overlapping spatial distribution. The clone represented by cluster 8 is confined to a single sampled branch of the glandular network.

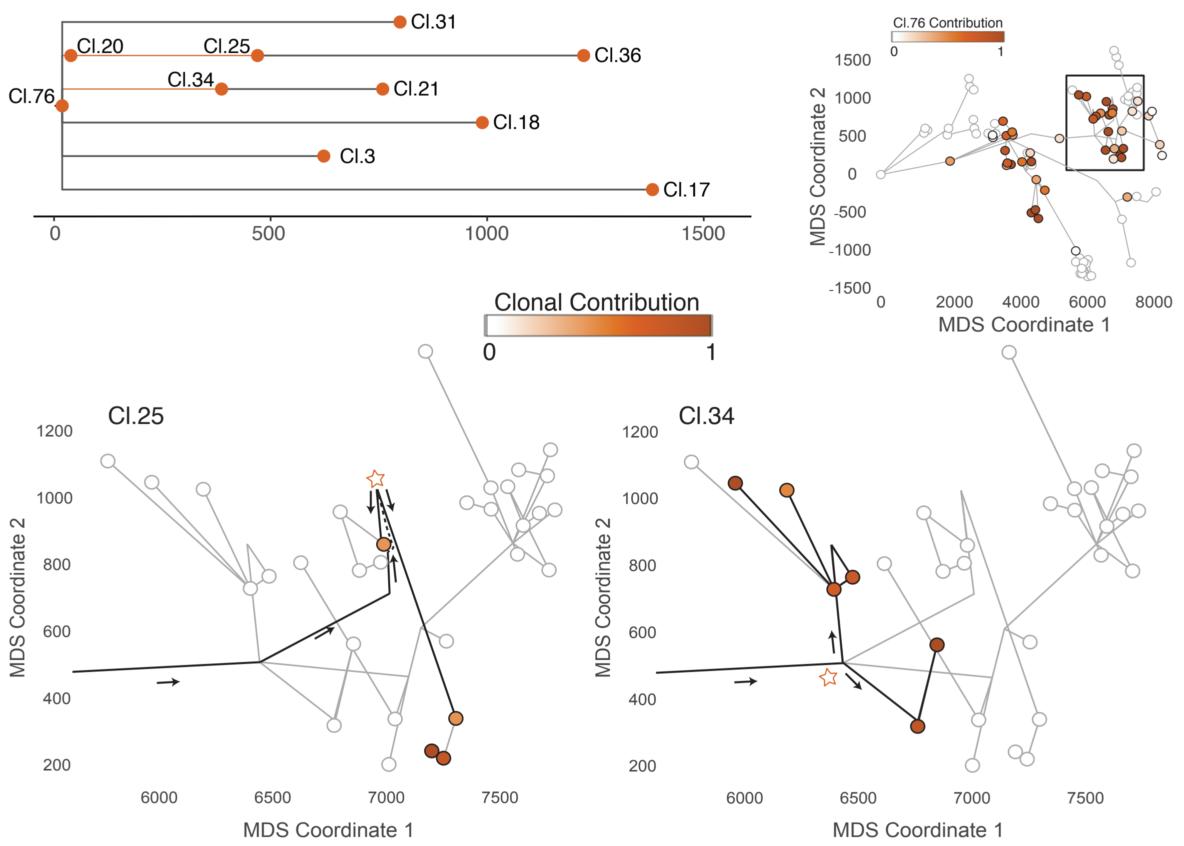

Supplementary Figure 4 (related to Figure 4): Cellular Contribution of Pubertal Clones Within the Ductal Subunit on the Left-Hand Side. The cellular contribution of pubertal clones represented by cluster 25 and 34 within the clonal territory of the embryonic clone represented by cluster 76 is highlighted. The proximal-to-peripheral direction of the ductal network is indicated by arrows. Both pubertal clones contribute to two distinct side-branches and their corresponding point from the main duct is highlighted with a star. Notably, in both cases the two branches seeded by the corresponding pubertal clones extend from the main duct directly on opposing sides and cannot be detected anywhere else. This strong spatial confinement suggests a localized progenitor cell before their respective pubertal expansions.

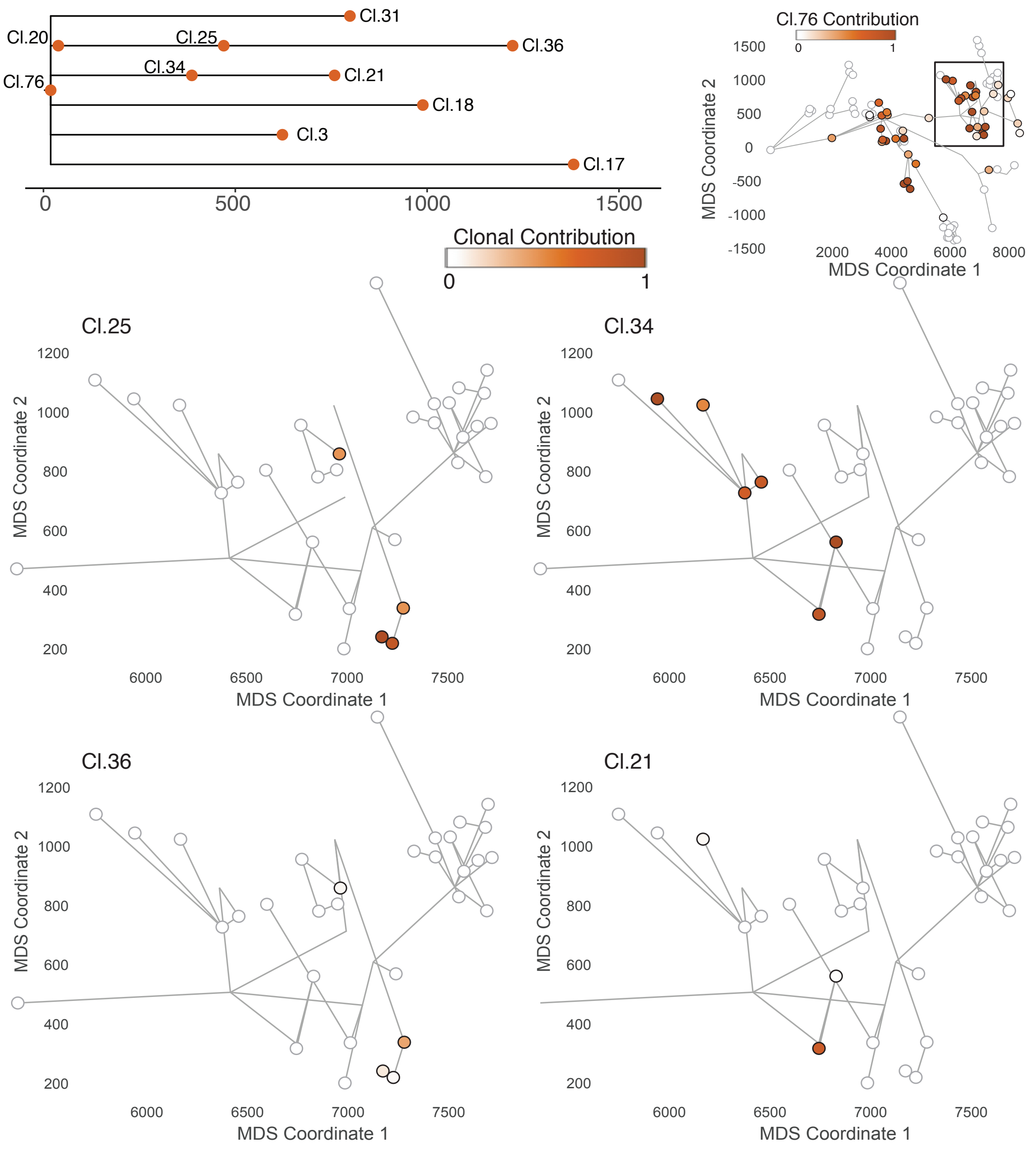
Cl.31
Cl.20
Cl.25
Cl.36
Cl.34
Cl.21
Cl.76
Cl.18
Cl.3
Cl.17
0
500
1000
1500
Cl.76 Contribution
MDS Coordinate 2
MDS Coordinate 1
Clonal Contribution
0
1
Cl.25
Cl.34
Cl.36
Cl.21

Supplementary Figure 5 (related to Figure 4): Embryonic, Pubertal and Adult Clones Occupy Increasingly Confined Territories. An example for embryonic, pubertal and adult clones within one clade of the left-hand side glandular subunit. The embryonic clone represented by cluster 76 occupies a wide spatial territory throughout most of the sampled branches. Pubertal clones represented by cluster 25 and 34 still display a confined spatial expansion with the embryonic territory. The adult clones represented by cluster 36 and 21 are only found within one or few microdissections overlapping their corresponding embryonic and pubertal clone. Notably, while most adult clones are found in peripheral microdissections, the highest cellular contribution of an adult clone is not necessarily associated with the most peripherally sampled microdissection as seen for both cluster 36 and cluster 21.

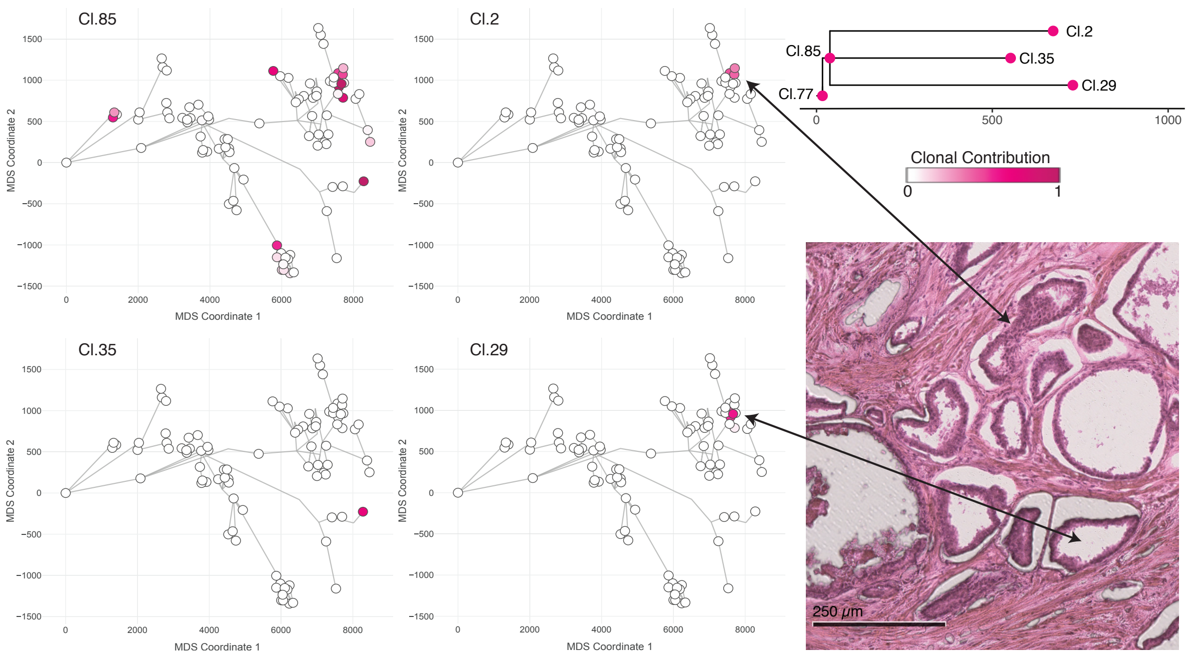

Supplementary Figure 6 (related to Figure 5): Phylogenetic Relatedness can be Independent of Spatial Proximity. An example for the glandular subunit of the left-hand side is shown. The three clones represented by cluster 2, 35 and 29 share a most recent common ancestor in the embryonic clone represented by cluster 85. Cluster 85 shows great spatial distribution across most sampled branches of the glandular subunit. While the clonal territories occupied by cluster 2 and 29 are spatially close as indicated by the structural overview as well as the histology image, the clonal territory occupied by cluster 35 is completely distinct. This example highlights the strongly localized nature of adult tissue maintenance that causes phylogenetic relationship to be largely independent of spatial proximity after embryonic and pubertal morphogenesis.

Supplementary tables

Table S1 (related to Figure 1): Overview of Available Prostate and WGS Samples. WGS from microdissections of histologically normal prostatic epithelium was obtained from eight different donors.

Donor ID	Sex	Donor Age [Years]	Sample Type	WGS Samples
PD43390	M	22	Post-mortem biopsy	32
PD43391	M	31	Post-mortem biopsy	9
PD43392	M	47	Post-mortem biopsy	2
PD37885	M	59	Post-mortem biopsy	5
PD40870	M	59	Whole prostate	319
PD42298	M	70	Biopsy	21
PD43393	M	71	Post-mortem biopsy	9
PD28690	M	78	Post-mortem biopsy	12

Table S2 (related to Figure 6): Overview of Non-synonymous Mutations in Significantly Mutated Genes in Prostate Cancer. Sixteen mutations called in microdissections from normal prostatic epithelium were found in previously published SMGs in prostate cancer. Most mutations were only called in a single microdissection.

Donor ID	Gene	Change	Impact	# of calls
PD42298	*KMT2A*	C1479W	Missense	1
PD42298	*ZNF292*	Y2193C	Missense	1
PD40870	*RPRD2*	D327G	Missense	1
PD40870	*RAG1*	R34W	Missense	1
PD40870	*RAG1*	V529M	Missense	1
PD40870	*MRE11A*	I647L	Missense	1
PD40870	*BRCA2*	F1336L	Missense	1
PD40870	*FOXA1*	R219S	Missense	5
PD40870	*NCOR1*	P1665T	Missense	1
PD40870	*NOX3*	V72I	Missense	1
PD40870	*CHD7*	R1155C	Missense	1
PD40870	*COL15A1*	A542D	Missense	2
PD40870	*AR*	.	Essential Splice	1
PD40870	*AR*	R832Q	Missense	1
PD40870	*SMARCA1*	W426*	Nonsense	1
PD28690	*PTPRC*	E362K	Missense	2
