# Supplementary material for: Development, maturation, and maintenance of human prostate inferred from somatic mutations
Source: Cell Stem Cell. 2021 Jul 1;28(7):1262–1274.e5. doi: 10.1016/j.stem.2021.02.005 (PMC8260206; doi:10.1016/j.stem.2021.02.005)
Supplement: Document S2. Article plus supplemental information [file mmc2.pdf]

Cell Stem Cell

Article

Development, maturation, and maintenance of human prostate inferred from somatic mutations

Graphical Abstract

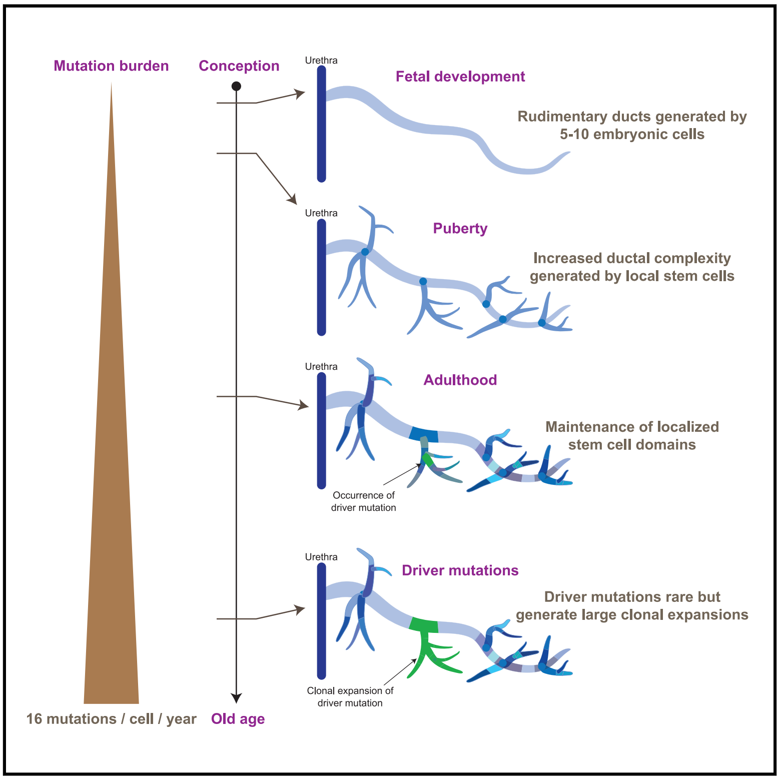

Highlights

- Somatic mutations accumulate with age in normal human prostate at ∼16/year/cell
- 5–10 embryonic cells found each of the 24–30 ductal subunits in fetal development
- Puberty acts on local stem cells in ductal units to increase branching complexity
- In adulthood, clones are small and spatially limited and have few driver mutations

Authors

Sebastian Grossmann, Yvette Hooks, Laura Wilson, ..., Michael R. Stratton, Rakesh Heer, Peter J. Campbell

Correspondence

rakesh.heer@newcastle.ac.uk (R.H.),
pc8@sanger.ac.uk (P.J.C.)

In brief

Using spontaneously occurring somatic mutations as lineage marks, Campbell, Heer, and colleagues define the stem cell dynamics of normal human prostate epithelium through fetal development, puberty, adulthood, and aging.

Grossmann et al., 2021, Cell Stem Cell *28*, 1262–1274
July 1, 2021 © 2021 The Authors. Published by Elsevier Inc.
https://doi.org/10.1016/j.stem.2021.02.005

CellPress

CellPress
OPEN ACCESS

Cell Stem Cell

Article

Development, maturation, and maintenance of human prostate inferred from somatic mutations

Sebastian Grossmann,[1] Yvette Hooks,[1] Laura Wilson,[2] Luiza Moore,[1] Laura O'Neill,[1] Iñigo Martincorena,[1] Thierry Voet,[1,3] Michael R. Stratton,[1] Rakesh Heer,[2,5,*] and Peter J. Campbell[1,4,5,6,*]
[1]Cancer Genome Project, Wellcome Trust Sanger Institute, Hinxton CB10 1SA, UK
[2]Translational and Clinical Research Institute, Faculty of Medical Sciences, Newcastle University, Newcastle, UK
[3]Department of Human Genetics, KU Leuven, 3000 Leuven, Belgium
[4]Wellcome – MRC Cambridge Stem Cell Institute, University of Cambridge, Cambridge CB2 0AW, UK
[5]Senior author
[6]Lead contact
*Correspondence: rakesh.heer@newcastle.ac.uk (R.H.), pc8@sanger.ac.uk (P.J.C.)
https://doi.org/10.1016/j.stem.2021.02.005

SUMMARY

Clonal dynamics and mutation burden in healthy human prostate epithelium are relevant to prostate cancer. We sequenced whole genomes from 409 microdissections of normal prostate epithelium across 8 donors, using phylogenetic reconstruction with spatial mapping in a 59-year-old man's prostate to reconstruct tissue dynamics across the lifespan. Somatic mutations accumulate steadily at ~16 mutations/year/clone, with higher rates in peripheral than peri-urethral regions. The 24–30 independent glandular subunits are established as rudimentary ductal structures during fetal development by 5–10 embryonic cells each. Puberty induces formation of further side and terminal branches by local stem cells disseminated throughout the rudimentary ducts during development. During adult tissue maintenance, clonal expansions have limited geographic scope and minimal migration. Driver mutations are rare in aging prostate epithelium, but the one driver we did observe generated a sizable intraepithelial clonal expansion. Leveraging unbiased clock-like mutations, we define prostate stem cell dynamics through fetal development, puberty, and aging.

INTRODUCTION

For an accessory sex gland about the size of a walnut, the prostate imparts a disproportionate burden of morbidity and mortality to the aging male. Prostate cancer is the most frequently diagnosed cancer in men and, in many countries, rivals lung cancer as the most common cause of male cancer death. Benign hyperplastic growth of the prostate, present almost universally to some degree in aging men, can cause symptomatic urinary tract obstruction requiring surgical or medical treatment.

The human prostate is distinctive for substantial morphogenic changes from fetal development through adolescence and into old age. The human prostate comprises approximately 24–30 independent glandular subunits, laid down *in utero*, extending from the urethra in a complex branching pattern before terminating in multiple acini (Aaron et al., 2016; McNeal, 1968; Moad et al., 2017). The branching glandular ducts are established in rudimentary form during embryonic development, with puberty causing a further burst of proliferation and neoformation that increases their convolution and complexity (Toivanen and Shen, 2017; Wang et al., 2001; Xue et al., 2001). During embryonic and pubertal development, ducts and acini form through elongation and branching of budding tips, which contain the progenitor cells responsible for directed morphogenesis (Cunha et al., 2018; Toivanen and Shen, 2017). Following these two phases of development, the prostate enters a relatively quiescent state of adult tissue maintenance. However, with increasing age, morphogenic activity restarts, causing neoformation of glandular tissue in the context of benign prostatic hyperplasia and prostate cancer (Cunha et al., 2018).

Proving the existence of adult stem cells in the human prostate has primarily relied on identification of populations expressing putative stem cell markers coupled with evaluation of their proliferative capacity *in vitro* and in xenografts (Collins et al., 2001; Goldstein et al., 2008; Hudson et al., 2000; Karthaus et al., 2014; Leong et al., 2008; Richardson et al., 2004). These studies suggest that the population of cells with stem-like properties is small and scattered throughout the glandular epithelium. This stem cell-like population is primarily located in the basal cell compartment, although recent studies have suggested that luminal cells also have considerable latent regenerative potential (Karthaus et al., 2020).

In mouse models, lineage tracing shows that rare prostate stem cells are capable of repopulating basal and luminal layers (Ousset et al., 2012; Wang et al., 2009). Nonetheless, differences between the human and mouse prostate mean that human studies are essential for understanding clinically relevant biology. To date, the most direct human data derive

 © 2021 The Authors. Published by Elsevier Inc.
This is an open access article under the CC BY license (http://creativecommons.org/licenses/by/4.0/).

CellPress
OPEN ACCESS

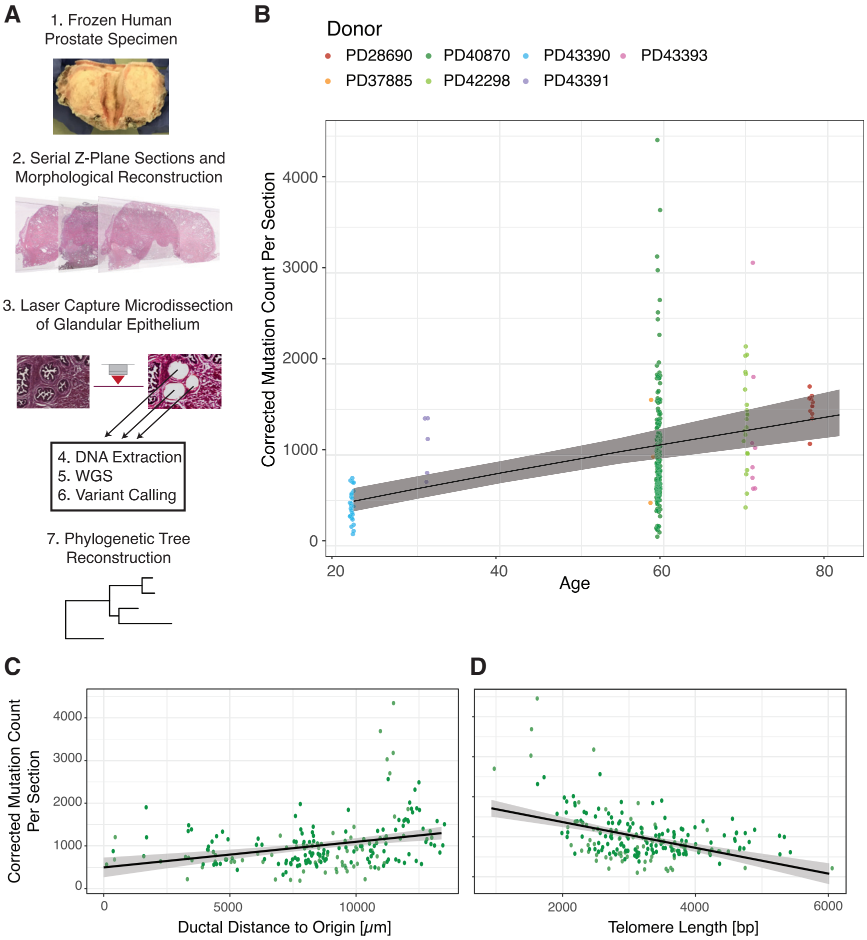

Figure 1. Whole-genome sequencing of targeted microdissections reveals dynamics of mutational burden in normal prostatic epithelium

(A) Workflow to uncover phylogenetic relationships in prostatic ductal tissue.

(B) The mutational burden in normal prostatic epithelium increases with age. Points represent the estimated mutation burden of individual microdissections rather than individual clones, colored by the donor from whom they derive. The line is fitted using linear mixed effects models, with shaded area showing the $CI_{95\%}$.

(C) Scatterplot showing the relationship between corrected mutation burden per microdissection (y axis) with distance from the urethral origin (x axis) for the 59-year old donor. The result remains statistically significant when outlying samples with a mutation burden of more than 2,000 are excluded. The R^2 for the regression model is 0.09.

(D) Scatterplot showing the relationship between corrected mutation burden per microdissection (y axis) with telomere length (x axis) for the 59-year old donor. The result remains statistically significant when outlying samples with a mutation burden of more than 2,000 are excluded.

WGS, whole-genome sequencing. The R^2 for the regression model is 0.20. See also Figure S1 and Table S1.

from studying mitochondrial DNA mutations in cytochrome *c* oxidase (COX) through *in situ* enzyme histochemistry (Blackwood et al., 2011; Gaisa et al., 2011; Moad et al., 2017). These studies establish a common clonal origin for basal, luminal, and neuroendocrine cells in human prostate epithelium, with some COX-deficient clones reaching all the way from urethra-proximal regions to distal acini (Moad et al., 2017). On this basis, it has been proposed that a stem cell niche is located proximal to the urethra, from which stem cells extend in directed streams to terminal acini (Moad et al., 2017).

Stem cell behaviors change through tissue development, adult homeostasis, and aging, which means that cellular dynamics and cell fate are difficult to unpick using clonal marks occurring early in life. Lineage tracing in mice and mitochondrial mutations in humans rely on a single clonal mark made in a cell at one point in time. Spontaneous somatic mutations, on the other hand, occur in all dividing cells throughout life, with studies across a range of epithelial tissues showing that this rate is largely constant across the human lifespan (Blokzijl et al., 2016; Brunner et al., 2019; Lee-Six et al., 2019; Martincorena et al., 2015, 2018; Moore et al., 2020; Yizhak et al., 2019; Yokoyama et al., 2019; Yoshida et al., 2020). We have previously exploited genome-wide spontaneous somatic mutations to study the population dynamics of blood stem cells (Lee-Six et al., 2018) but have not applied such a methodology to assess stem cell turnover in a solid tissue such as the human prostate. To achieve this, we undertook extensive whole-genome sequencing to define clonal relationships among epithelial cells, combining this with detailed morphological reconstruction of the organ to understand spatial patterning of clones.

RESULTS

Spatially resolving the clonal dynamics of human prostate epithelium

We applied laser-capture microdissection to isolate samples of 200–500 contiguous prostate epithelial cells from eight donors ranging from 22–78 years of age (Figure 1A; Table S1). Given that somatic mutations accumulate from conception, we were particularly interested in exploring the clonal dynamics of normal prostate epithelium through development, maturation, and adult tissue maintenance. To this end, we focused our studies predominantly on the normal prostate of a 59-year donor who had a radical cystoprostatectomy for micropapillary variant bladder cancer, stage pT1. The donor was of European ancestry with no family history of prostate cancer. The prostate-specific antigen (PSA) level was 1.8, and a clinically benign prostate was noted. The prostate size was 30 cm^3, and histologically, there was no evidence of benign prostatic hyperplasia, adenomas, prostatitis, or adenocarcinoma.

We obtained serial sections through the whole intact prostate gland, staining and imaging 671 slides of 10-μm depth. From these images, we morphologically reconstructed two entire

ductal units from their proximal urethral outlet back through progressive branchpoints to their terminal acini. Using multiple laser-capture microdissections, we isolated contiguous populations of ~200–500 epithelial cells from ducts and acini across the full span of the two ductal units, performing whole-genome sequencing on them. We reconstructed phylogenetic lineage trees from the somatic mutations and layered these onto reconstructions of the physical ductal structures to inform models of clonal dynamics.

The final dataset comprised 409 whole genomes, sequenced to typical depths of 25–35× (Figures S1A–S1C), across the 8 donors. This included 319 whole genomes from the 59-year-old man's prostate that were studied most intensively. Somatic mutations were identified from each genome; the observed variant allele fraction for mutations typically fell in the range of 0.15–0.3 (Figure S1B), suggesting that 30%–60% of cells within a given microdissection were clonally related.

Somatic mutations accumulate with age

Across the cohort, the burden of somatic mutations in each microdissection increased with age (Figure 1B). To formally assess this relationship, we corrected the observed mutation burden for detection sensitivity based on sequencing depth and variant allele fraction, excluding 31% of samples where the coverage or clonality were too low to accurately estimate sensitivity (STAR methods). Although this will mostly correct for the relationship of sensitivity to depth and variant allele fraction, we acknowledge that complex clonal structure and very low-frequency clones will lead to some inaccuracy. We used linear mixed effects models to estimate the rate of mutation accumulation while accounting for within-donor correlation structure. We found a strongly significant effect of age, corresponding to a mutation rate of 16.4 mutations per year per clone (95% confidence interval [$CI_{95\%}$] = 9.7–23.2, p = 0.004). The relationship was linear across the age range studied here, with the intercept not significantly different from zero (intercept = 133 mutations, $CI_{95\%}$ = −262 to 528). These data suggest that the somatic mutation rate in prostate epithelium is likely constant throughout life and certainly in the adult age range studied here. A genome-wide mutation burden of ~1,000–1,500 mutations by 60–80 years of age is within range of the median burden observed in adenocarcinoma of the prostate (ICGC/TCGA Pan-Cancer Analysis of Whole Genomes Consortium, 2020), suggesting that prostate cancers do not always have elevated rates of base substitutions compared with normal prostate epithelium.

We observed substantial variation in mutational burden of microdissections, with the within-donor standard deviation estimated to be 550 mutations/sample from the mixed effects models (Figure 1B). Indeed, 9 microdissections had more than 2,000 mutations, samples from different regions of the prostate with no apparent technical reasons for their elevated burden. The observed spectrum and inferred signatures of somatic mutations in normal prostate epithelium were consistent with endogenous, clock-like mutational processes (Figures S1D and S1E). In 399 of the 409 microdissections, mutations could be entirely attributed to the clock-like signatures SBS1, SBS5, and SBS40; these are mutational signatures that are ubiquitous and increase linearly with age across normal tissues and cancers (Alexandrov et al., 2015, 2020; Blokzijl et al., 2016; Lee-Six et al., 2019; Moore et al., 2020; Yokoyama et al., 2019; Yoshida et al., 2020). Interestingly, unlike other tissues where endogenous mutational processes predominate, such as colon or endometrium, prostate epithelium has much greater within-donor variation in mutation burden.

Insertions or deletions (indels) were approximately 10-fold less frequent than base substitutions (Figure S1F); copy number changes and structural variants were very rare (Figure S1G). We found an example of a chromothripsis event affecting chromosome 9q in a single microdissection (Figure S1H), echoing similar rare instances of chromothripsis seen in normal liver and lung clones (Brunner et al., 2019; Yoshida et al., 2020).

The mutation burden increases from the urethra to peripheral zones

To assess whether the striking within-donor variation in mutation burden could be explained by spatial effects, we generated high-resolution maps of entire glandular subunits in a 59-year-old prostate (PD40870). The mutation burden increased significantly in the proximal-to-distal direction within branching ductal trees from the urethra into the periphery of the prostate (0.06 mutations/μm, $CI_{95\%}$ = 0.03–0.09, R^2 = 0.09, $p < 8 \times 10^{-6}$; Figure 1C). The progressive mutational burden in the periphery of the prostate gland was independent of clonality, which remained constant along the lengths of glandular subunits (Figure S1I). We saw no differences in mutation signature by distance from the urethra, suggesting that the spatial variation in burden arose from variable activity per unit time of the same clock-like mutational processes.

A possible explanation for a proximal-to-distal gradient of mutations arising through endogenous processes would be that distal epithelial cells had undergone more cell divisions than proximal cells. To assess this, we estimated telomere lengths from the whole-genome sequences (Farmery et al., 2018). We found that, on average, telomere length decreased as distance from the urethra increased (−0.08 bp/μm, $CI_{95\%}$ = −0.05 to −0.12, $p = 1 \times 10^{-5}$) and decreased similarly with higher mutational burden (−0.32 bp/mutation, $CI_{95\%}$ = −0.23 to −0.41, R^2 = 0.20, $p < 1 \times 10^{-10}$; Figure 1D).

These results suggest that mutation accumulation, cell division, and histological location are tightly interlinked in normal prostatic epithelium. Accumulation of mutations increases significantly in a proximal ductal-to-distal acinar direction, associated with progressive shortening of telomeres, indicating that cells in the peripheral prostate have undergone higher numbers of previous cell divisions. This is of particular relevance to prostate cancer, which occurs predominantly in the peripheral gland. Nonetheless, there remains much residual, unexplained variation in mutation burden, suggesting that other unknown factors also play an important role in mutation accumulation.

Complex lineage relationships within glandular subunits

Information about somatic mutations can be used to infer lineage relationships among somatic cells. With single-cell-derived data, lineage trees can be inferred directly from a matrix of mutation identity across cells (Behjati et al., 2014; Lee-Six et al., 2018; Woodworth et al., 2017). However, our microdissections from prostate epithelium comprised a few hundred to thousand somatic cells, which means that clonal mixtures can be present

CellPress
OPEN ACCESS

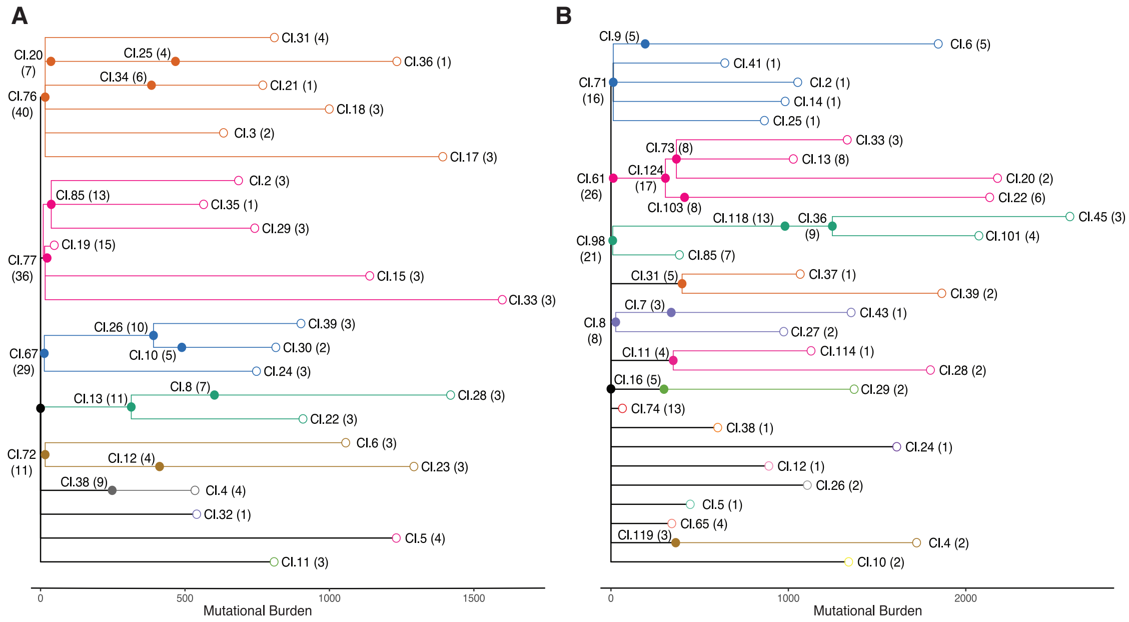

Figure 2. Phylogenetic trees of prostatic epithelium from two distinct glandular subunits

Ancestral clones that gave rise to the sampled microdissections were arranged in a lineage tree based on their co-occurrence pattern and cellular contribution to individual microdissections. The number of microdissections in which the corresponding ancestral clone contributed to at least 10% of the microdissected cells is indicated in brackets. Lengths of branches (x axis) indicate the numbers of mutations assigned to that branch. Closed circles represent coalescent events; open circles represent the location of the terminal tip of each branch. Coloring of clades is according to descendants of different embryonic cells.

(A) Phylogenetic tree for a glandular subunit on the left side of the prostate.

(B) Phylogenetic tree for a glandular subunit on the right side of the prostate.

See also Figure S2.

in the genome data from a single sample. We therefore adapted methods from cancer genomics to cluster mutations and reconstruct phylogenetic trees (Brunner et al., 2019; Nik-Zainal et al., 2012b), focusing on the two extensively sampled ductal units from the 59-year-old prostate (PD40870). The two ductal subunits occupied distinct regions in the left and right sides of the prostate and will be referred to as such hereafter.

Our algorithm identifies clusters of individual mutations that have concordant distribution of variant allele fractions across the many microdissections. A handful of clusters, up to 10 per ductal unit, contained mutations present at high allele fraction in multiple microdissections; such clusters tended to contain small numbers of mutations (Figure S2). These clusters define clades near the root of the phylogenetic trees. Further clusters of mutations tended to have more mutations but were represented in fewer microdissections and often at lower mean variant allele fraction. Importantly, a nesting pattern was evident among clusters, defining clear temporal lineage relationships across sets of mutations. As a result, we were able to reconstruct phylogenetic trees reflecting the entire dataset with minimal ambiguity.

Phylogenetic trees for the two morphologically reconstructed ductal units comprised a total of 37 and 43 clones from the left and right units, respectively (Figure 2). The 37 clusters from the left glandular structure were divided into nine distinct clades defined by early branches (Figure 2A), four of which encompassed 28 of the 37 mutation clusters and featured branching and nested relationships. Likewise, most of the 43 clones in the right unit were distributed across 5–6 clades defined by early branches (Figure 2B), again exhibiting branching and nested clonal relationships.

Two waves of lineage coalescences during embryogenesis and puberty

Branchpoints in phylogenetic trees, formally known as coalescences, represent past cell divisions when considering somatic lineage trees. Because our data show that prostate tissue, like other normal tissues, accumulates mutations linearly with age, we can use this rate as a molecular clock to estimate the chronological age of key lineage-defining events.

The left and right ductal units showed large clades defined by very few mutations (Figure 2). For example, on the left, there were four clusters, each containing only 13–16 mutations, which defined clades containing terminal branches extending to approximately 1,000–1,500 mutations. Similar patterns were present in the right unit, with four clusters, each containing fewer than 20 mutations, accounting for the majority of microdissections. With an estimated mutation rate of 10–20 mutations per year in the prostate, clones defined by fewer than 20 mutations likely represent ancestral cells that existed very early in life, presumably during fetal development. The high density of coalescences (filled circles in Figure 2) within the first 20 mutations of molecular time confirms a rapid increase in clonal complexity of the developing prostate during embryogenesis.

After these early branchpoints, a second wave of coalescences is evident in both ductal units at around 300–500

mutations of molecular time. For example, on the left, 8 coalescence events were observed in 5 clades with an average total branch length of 414 mutations (SD = 117; range, 248–602; Figure 2A). On the right, a comparable increase in the density of coalescences occurred at a similar stage in molecular time, when the ancestral cells had an average burden of 338 mutations (SD = 66; range, 193–415; Figure 2B).

Using the estimates of mutation rate from our regression model, we calculate that an average burden of 300–400 mutations is reached by a prostate cell between the ages of 10 and 17 years (based on an intercept of ~130 mutations and a rate of 16.4 mutations/year). We therefore infer that this second wave of coalescences occurs during puberty.

After these two waves of coalescences, we observe very few branchpoints in the phylogenetic trees. This suggests that cellular turnover during adulthood is largely defined by tissue maintenance rather than ongoing physical expansion of clones.

Morphogenesis during fetal development

One of the key advantages of our intensive sampling strategy combined with detailed morphological reconstruction of the glandular subunits is that we can layer information gleaned from the phylogenetic tree onto the physical ductal tree. More explicitly, for each cluster of mutations, we know which microdissections carried those mutations and in what fraction of cells within each microdissection as well as the ductal connections among those microdissections. This enables us to define the current geographical scope achieved by each of the ancestral clones. Here we used multidimensional scaling to collapse their physical positions into two dimensions for plotting, but three-dimensional interactive graphics can be explored online (https://sg18.shinyapps.io/RightStructure_TreeAnd3DModel/ and https://sg18.shinyapps.io/LeftStructure_TreeAnd3DModel/).

We first evaluated the physical distribution of clusters of mutations that occurred during embryogenesis, within the first 20 mutations of molecular time (Figure 3; Figure S3). We observed 5 embryonic clusters in the right glandular subunit and 7 embryonic clusters on the left that displayed a wide geographical distribution across the main gland trunk and its branching ducts. The general pattern of these embryonic clones is one in which each clone contributes to several large contiguous areas of the ductal subunit. Many of these contiguous areas span the main duct and several secondary ducts, often accounting for tens of the microdissections we sequenced. However, for a given embryonic clone, there are also large, interspersed regions of the ductal subunit where that clone makes zero contribution, leading to a patchwork, mosaic distribution of involvement.

Several microdissections showed a mixed contribution from more than one embryonic clone (some examples are indicated by arrowheads in Figure 3). We found that the occurrence of microdissections derived from multiple independent embryonic clones did not significantly correlate with distance from the urethra; even some terminal acini showed multiclonal contributions. These microdissections comprising more than 10% contribution from more than one embryonic clone were a minority overall, representing 27% (47 of 177) of samples.

Our data suggest that each of the 24–30 ductal units present in a human prostate is rapidly laid down during embryogenesis by as few as 5–10 individual ancestral cells. The contributions of these embryonic cells to the ductal tree represent a clonal patchwork; sometimes large, connected sections of the tree entirely derive from a single embryonic cell, sometimes embryonic clones show admixture in individual acini or ducts, and sometimes a given embryonic clade will generate multiple discontinuous regions of the ductal tree.

Further increase in ductal complexity at adolescence

We next studied the geographical distribution of clones that were active during adolescence, defined by branchpoints in the phylogenetic tree at around 300–500 mutations. Although clones that existed during embryonic development were distributed widely across several main and side branches of the ductal network, clones associated with pubertal maturation were found in one or a few directly adjacent side branches (Figure 4; Figure S4).

In one example from the right ductal unit, we observed three coalescences from the same embryonic clade, each timed to adolescence and occurring within 50 mutations of molecular time of one another (Figure 4). Descendants from the most ancestral of the pubertal coalescences, cluster 124, populated 3–4 terminal branches within the ductal unit, spanning 18 of our microdissections. From the phylogenetic tree, this clone split into two subclones, defined by mutations in clusters 73 and 103, also timed to adolescence. These two subclones occupied mutually exclusive regions of the prostate, accounting for neighboring but distinct terminal branches of the ductal unit. The mutations that mark each of the pubertal subclones were present at high variant allele fractions within their respective terminal branches, indicating that the vast majority of epithelial cells in this region were descended directly from the pubertal subclones.

We also found several examples of points where two minor tributary sub-branches fed into a main duct at the same location (marked with a star in Figure S4). Interestingly, in these cases, a clone present at adolescence seeded both tributary branches but made no contribution to branches farther down the main duct or the main duct itself. Again, the mutations that defined the pubertal subclones were present at high variant allele fraction in these tributary branches, suggesting that here the entirety of the epithelium derived from these ancestral cells. This is consistent with creation of side branches at puberty by a stem cell clone initially disseminated to that region during fetal development.

These data suggest that puberty drives further remodeling of the prostate gland by local and rapid expansion of epithelial progenitors already scattered through the ductal tree, generating new side and terminal branches.

Adult prostate tissue maintenance

With one exception, discussed further in the next section, we observed no coalescences in the phylogenetic tree that could be timed to adulthood. Instead, clusters with many hundreds of mutations acquired after puberty were evident as long terminal branches in the phylogeny. Mutations acquired during adulthood were typically restricted to fewer than 5 microdissections in direct anatomical proximity (Figure 5; Figures S5 and S6).

Most of the terminal nodes of the phylogenetic tree marking the period of adult tissue homeostasis were spatially confined to regions spanning a few hundred micrometers and

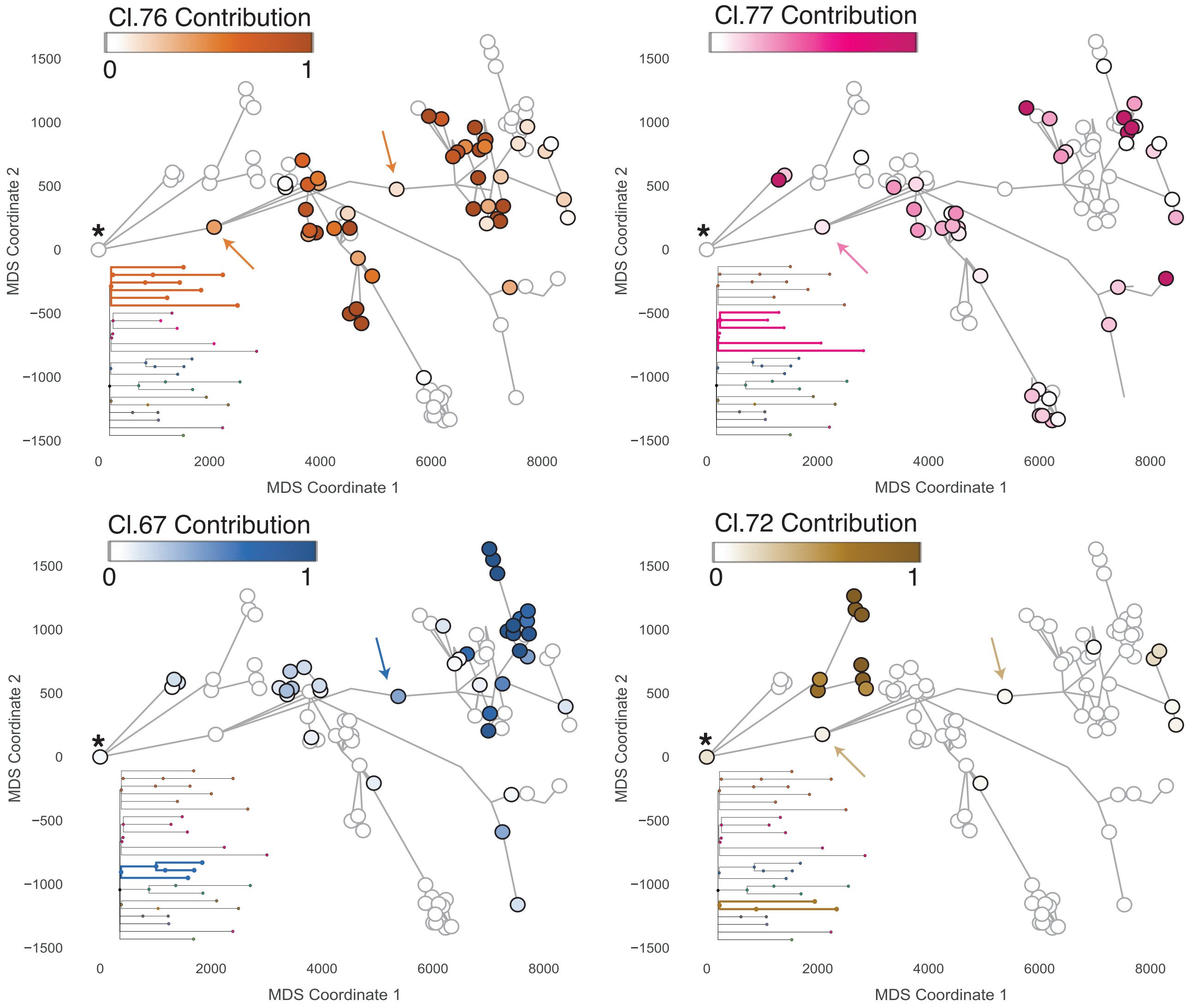

Figure 3. Distribution of embryonic clones mirrors ductal morphogenesis

The cellular contribution of four ancestral clones from embryonic development is displayed for the glandular subunit from the left side. Each circle marks a microdissection, and the ductal connection is indicated by gray lines. Microdissections with positive contribution from the clone are circled in black, whereas those with zero contribution are circled in gray. The most urethra-proximal microdissection is marked with an asterisk. The four embryonic clones display a wide and mainly contiguous spatial distribution. Although their general distribution overlaps, the contribution to individual microdissections is largely mutually exclusive. Two examples of microdissections with contribution from multiple clones are marked with arrows. See also Figure S3.

independently acquired hundreds of private mutations. This suggests that, during adulthood, prostate epithelium retains a high degree of spatial organization with minimal migration. Cell turnover occurs locally, with the consequence that there are many independent clonal territories maintained by individual stem and progenitor cells distributed throughout the ductal tree.

Driver mutations are rare in normal prostate but can trigger clonal expansion

We found only two coalescent events likely to have occurred in adulthood, both within the same clade of the right ductal tree (Figure 6A). Cluster 118 contained 981 mutations, which subsequently seeded a subclone containing 1,248 mutations (cluster 36), burdens suggesting that this clonal diversification took place when our donor was in his 30s or 40s. In contrast to the limited spatial distribution of other adult clones, mutations from cluster 118 could be identified across 13 microdissections covering one main peripheral duct with four terminal branches. The cellular contribution of mutations from cluster 118 ranged between 40% and 80% across these microdissections. The subclone marked by mutations in cluster 36 distributed over a similar geographic range, albeit at somewhat lower cellular fractions. The histological appearance of epithelium deriving from this clone was normal (Figure 6B), suggesting that the driver mutation was not sufficient to transform the cells.

This clone was notable for an R219S mutation in the *FOXA1* gene assigned to cluster 118. This gene is mutated in ∼10% of prostate cancers, typically at hotspots within the forkhead

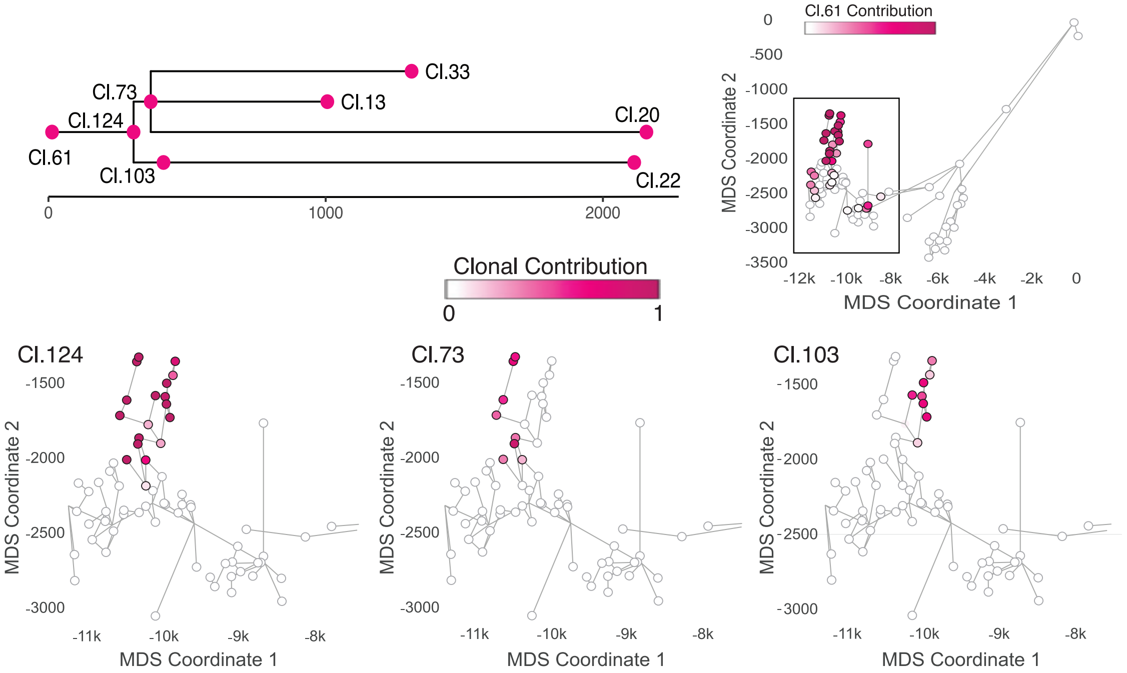

Figure 4. Localized expansion of adolescent clones marks increased ductal complexity during puberty

The cellular contribution to the right structure from ancestral clones 73, 103, and 124 that are associated with puberty is displayed. The physical location of each microdissection has been collapsed into two dimensions using multidimensional scaling, each marked with a circle. Ductal connections between microdissections are illustrated with straight black lines. The circle representing each microdissection is colored according to the fraction of cells in that sample that derive from the particular adolescent clone. Microdissections with positive contribution from the clone are circled in black, whereas those with zero contribution are circled in gray. See also Figures S4 and S5.

DNA-binding domain, including the R219 residue (Adams et al., 2019; Cancer Genome Atlas Research Network, 2015; Gerhardt et al., 2012). Such a canonical hotspot driver mutation in the only clone we found to have undergone sizable expansion during adulthood argues that driver mutations disrupt the normal, tightly controlled balance of adult tissue homeostasis.

We found no other known driver mutations in our dataset of 409 whole genomes. In particular, we found none of the *ERG*-family fusion genes or structural variants, no *SPOP* mutations, no further *FOXA1* mutations, and none of the recurrent whole chromosomal aneuploidies seen in prostate adenocarcinoma (Armenia et al., 2018; Attard et al., 2016; Gasi Tandefelt et al., 2014). Furthermore, methods for *de novo* discovery of significantly mutated genes based on an excess of non-synonymous mutations revealed no significant hits (Martincorena et al., 2017), even when hypothesis testing was restricted to known prostate cancer genes (Armenia et al., 2018; Table S2).

DISCUSSION

Our data demonstrate the insights into development and maintenance of solid tissue organs in humans that can be obtained using spontaneously occurring somatic mutations as a molecular clock. For all organ systems studied to date, the burden of somatic mutations from endogenous mutational processes increases linearly across the human lifespan (Alexandrov et al., 2015; Blokzijl et al., 2016; Brunner et al., 2019; Franco et al., 2018; Lee-Six et al., 2018; Lee-Six et al., 2019; Martincorena et al., 2018; Moore et al., 2020); here, we show that this includes the prostate gland. With an estimated mutation rate of 16 mutations per clone per year, a new clonal mark is laid down every 3 weeks or so, on average, in each stem or long-lived progenitor cell in prostate epithelium. Coupled with high-resolution spatial mapping of clones, this enables unbiased, quantitative lineage tracing of cell fate throughout the lifespan of human tissue that is otherwise only achievable in genetically manipulated experimental animal models.

The model that emerges for the human prostate gland is of two major waves of ductal morphogenesis: during embryogenesis and puberty, followed by limited clonal expansion during adulthood (Figure 7). We estimate that each of the 24–30 individual ductal units draining into urethra can be generated by as few as 5–10 embryonic cells; these are responsible for invading the surrounding urogenital sinus mesenchyme, establishing the branching structure and populating that duct with epithelial cells. With branching into progressively smaller ducts, the 5–10 founder cells become increasingly segregated; this manifests as a mixed clonal population near the urethra, with relative clonal purity more distally, consistent with the streaming appearance seen with loss-of-function COX mutations (Moad et al., 2017)

Puberty drives further morphogenesis in the human prostate gland, with histological studies showing that new side branches and terminal acini are created, substantially increasing the surface area of secretory epithelium (Cunha et al., 1987; Wang et al., 2001; Zaichick and Zaichick, 2013). Our data show that this additional morphogenesis is undertaken by local progenitor/stem cells

CellPress
OPEN ACCESS

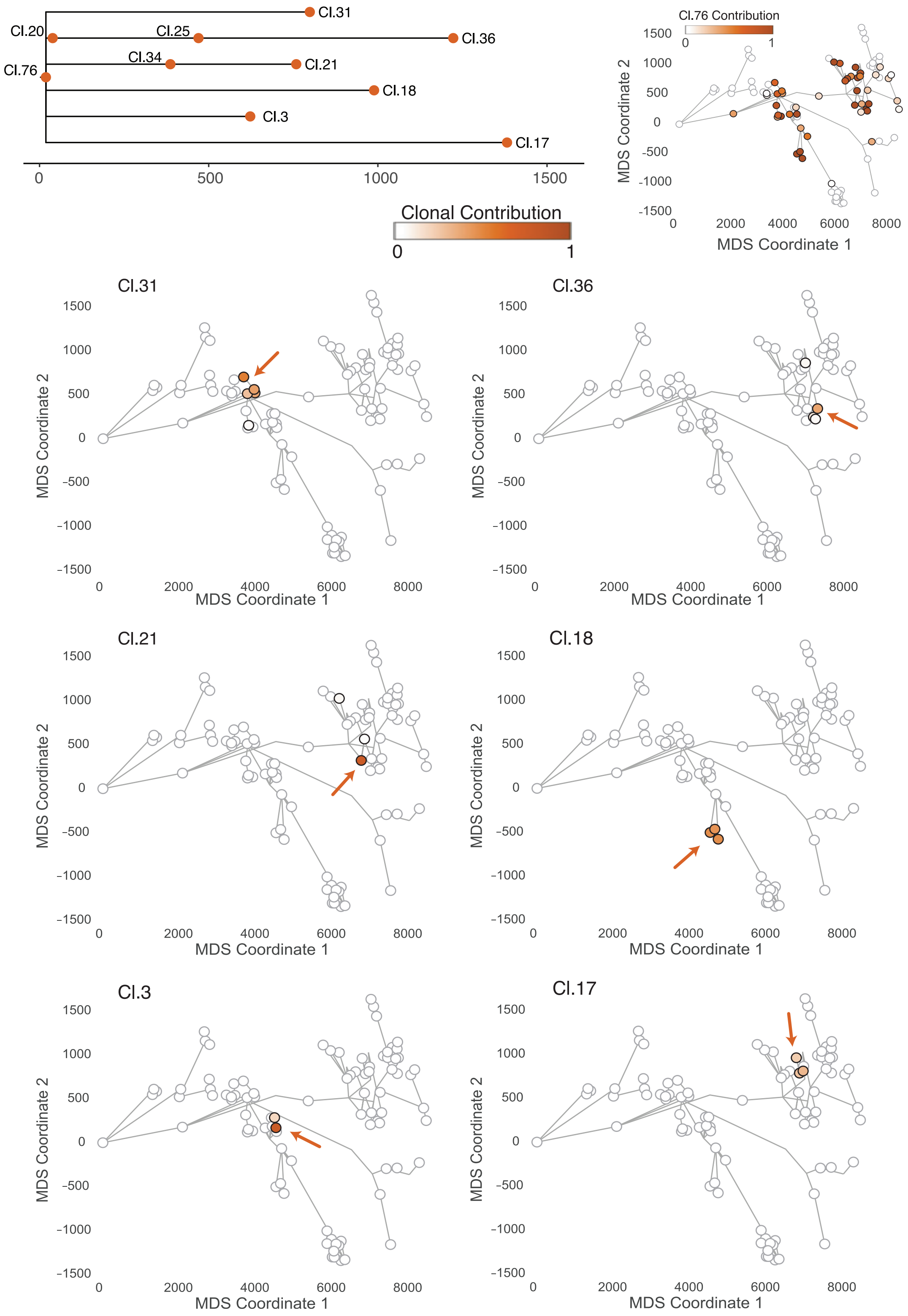

Figure 5. Ancestral clones marking adult tissue homeostasis are spatially confined

The distribution of six clones dated to adult tissue maintenance of the left glandular subunit is displayed. The location of individual ancestral clones is highlighted with an arrow. All adult clones can be found in the corresponding embryonic territory. The embryonic clone represented by cluster 76 simultaneously marks the most recent common ancestor of the six adult clones displayed. Microdissections with positive contribution from the clone are circled in black, whereas those with zero contribution are circled in gray. See also Figure S6.

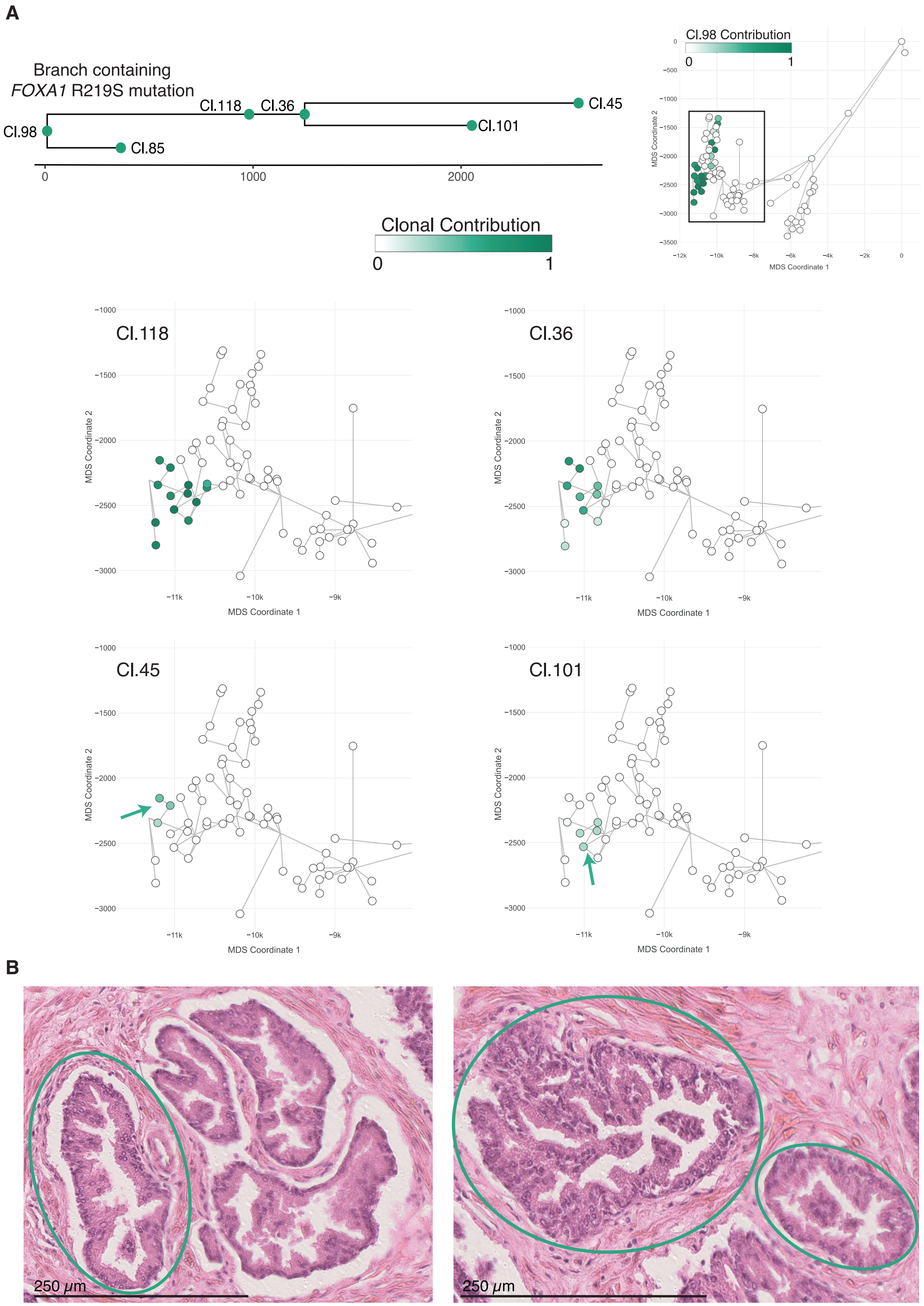

Figure 6. Rare driver mutations trigger clonal expansion in normal prostatic epithelium

(A) The R219S prostate cancer driver mutation in *FOXA1* was detected in the right glandular subunit within the ancestral clone that is represented by cluster 118. Three additional clusters dating to adulthood were nested under cluster 118. This was the only detected example of sub-nesting of ancestral clones that must

(legend continued on next page)

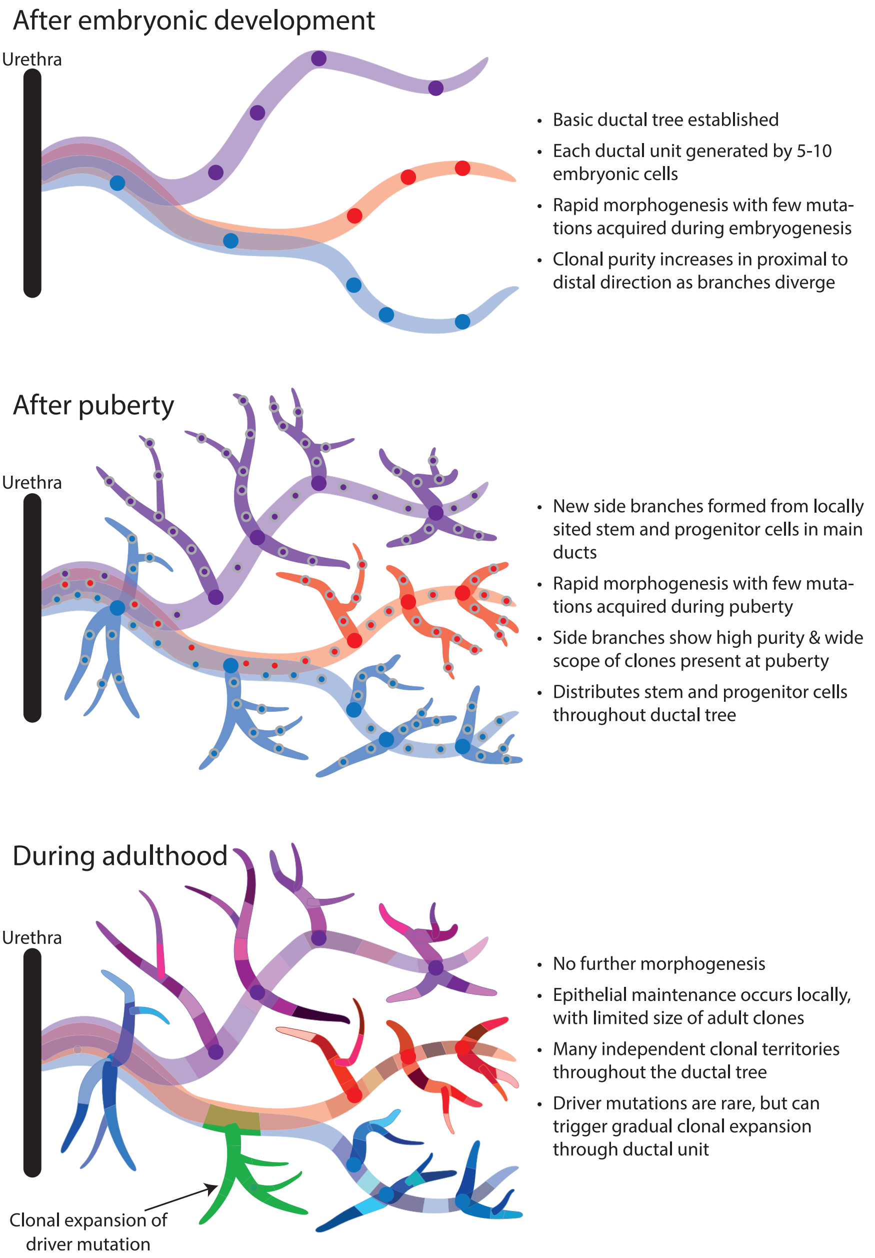

Figure 7. Model of clonal dynamics in normal prostatic epithelium

After adolescence, the prostate enters a relatively quiescent stage of adult tissue maintenance. We find that clones dated to adulthood are spatially constrained, with limited migration; significant cellular contribution from a given set of mutations acquired in adulthood usually only spans one or few adjacent microdissections separated by less than 1 mm physical distance. We found only one driver mutation across two entire ductal units (collectively representing 5%–8% of the prostate), suggesting that driver mutations occur at 1–2 orders of magnitude lower frequency than in normal skin, esophagus, lungs, and endometrium (Martincorena et al., 2015, 2018; Moore et al., 2018; Suda et al., 2018; Yizhak et al., 2019; Yokoyama et al., 2019; Yoshida et al., 2020), more in keeping with the low rates seen in the colon or liver (Brunner et al., 2019; Lee-Six et al., 2019). The one canonical hotspot mutation we did observe, however, in *FOXA1*, drove a clonal expansion encompassing one main peripheral duct and four terminal branches, considerably larger than the scope of any other clone from adulthood. This expansion echoed those seen earlier in life, consistent with the theory of "embryonic reawakening" proposed for prostate cancer and benign prostatic hyperplastic growth.

These data have interesting implications for how prostate cancer develops. We find that local stem and progenitor cells acquire many hundreds, sometimes thousands, of private mutations across a lifetime. The highest mutation rates and greatest telomere attrition occur in peripheral regions of the prostate, where cancer incidence is highest. Low turnover and limited post-pubertal migration mean that these stem and progenitor cells remain segregated, evolving independently, explaining why physically separated foci of cancer in the same man's prostate tend to be clonally unrelated (Boutros et al., 2015; Boyd et al., 2012; Cooper et al., 2015; Lindberg et al., 2013). The rare normal clone with a driver mutation can take decades to expand, tracking and respecting the

seeded throughout the ductal tree during embryogenesis, which are reactivated by the burst of androgens in puberty. This is evidenced by the observation that entire side and terminal branches derive from a single most recent common ancestor whose existence can be dated to puberty. Moreover, mutations acquired by each of those clones between completion of fetal development and puberty cannot be detected anywhere outside of the specific side branches or terminal units they founded.

have existed during adult tissue maintenance. Microdissections with positive contribution from the clone are circled in black, whereas those with zero contribution are circled in gray.
(B) Exemplary histology of microdissections with the *FOXA1* driver mutation. Epithelial structures enclosed in green circles were subjected to WGS and contained the R219S prostate cancer driver mutation in *FOXA1*. Additional visible structures were not sequenced. Despite the known cancer driver mutation, all epithelial structures are within normal histological limits for an aging prostate.
See also Table S2.

complex ductal structure laid down in development and puberty; this provides a long window for early detection and intervention.

Limitations of study

Our study relies on microdissection and sequencing of small numbers (200–500) of epithelial cells, with the potential that individual samples can represent a mixture of clones. This limits our ability to detect mutations present in a small fraction of cells within the microdissection, with the corollary that our calculations of mutation burden are imperfect estimates. Single-cell methods, especially sequencing single-cell-derived organoid cultures (Drost et al., 2016), would allow more accurate estimates of mutation burden. Combining organoid derivation with lineage tracing using spontaneous somatic mutations could also enable evaluation of the dynamics of commitment of stem cells to luminal and basal phenotypes.

We note that our study focused predominantly on a single 59-year old healthy subject. Future studies using these methods generate should widen the sample size, including a broader age range, individuals with benign prostatic hyperplasia, and individuals with prostate neoplasia, especially early disease.

STAR★METHODS

Detailed methods are provided in the online version of this paper and include the following:

- KEY RESOURCES TABLE
- RESOURCE AVAILABILITY
  - Lead contact
  - Materials availability
  - Data and code availability
- EXPERIMENTAL MODEL AND SUBJECT DETAILS
- METHOD DETAILS
  - Sample Preparation for Laser-Capture Microscopy
  - Reconstruction of Prostatic Glandular Subunits
  - Laser-Capture Microscopy and Whole-Genome Sequencing
  - Genome Alignment and Variant Calling
  - Inference of the Relationship Between Mutation Accumulation and Aging
  - Mutational Spectra and Signature Extraction
  - Detection of Positive Selection and Driver Mutations
  - Telomere Length Estimation
  - Lineage Tree Inference
- QUANTIFICATION AND STATISTICAL ANALYSES
- ADDITIONAL RESOURCES

SUPPLEMENTAL INFORMATION

Supplemental Information can be found online at https://doi.org/10.1016/j.stem.2021.02.005.

ACKNOWLEDGMENTS

This work was supported by the Wellcome Trust. R.H. acknowledges further support through a PCF Challenge Research Award (18CHAL11). We thank the Sanger Institute staff of WTSI Sample Logistics, Sequencing and Informatics for their contribution and all individuals who contributed samples to this study.

AUTHOR CONTRIBUTIONS

S.G., R.H., and P.J.C. conceived and supervised the project. L.O. managed sample storage, processing, and administrative tasks. L.M. and R.H. provided histological guidance. L.M., L.W., S.G., and Y.H. carried out laboratory work. S.G. performed bioinformatic analyses. P.J.C., R.H., and S.G. interpreted the results and derived the proposed model of prostate stem cell dynamics. I.M., M.R.S., and T.V. provided critical feedback and helped with interpretation of the results. P.J.C., R.H., and S.G. wrote the manuscript. All authors read and approved the final manuscript.

DECLARATION OF INTERESTS

P.J.C. is a founder, consultant, and stockholder of Mu Genomics Ltd.

Received: July 5, 2020
Revised: November 19, 2020
Accepted: February 2, 2021
Published: March 2, 2021

REFERENCES

Aaron, L., Franco, O.E., and Hayward, S.W. (2016). Review of Prostate Anatomy and Embryology and the Etiology of Benign Prostatic Hyperplasia. Urol. Clin. North Am. *43*, 279–288.

Adams, E.J., Karthaus, W.R., Hoover, E., Liu, D., Gruet, A., Zhang, Z., Cho, H., DiLoreto, R., Chhangawala, S., Liu, Y., et al. (2019). FOXA1 mutations alter pioneering activity, differentiation and prostate cancer phenotypes. Nature *571*, 408–412.

Alexandrov, L.B., Jones, P.H., Wedge, D.C., Sale, J.E., Campbell, P.J., Nik-Zainal, S., and Stratton, M.R. (2015). Clock-like mutational processes in human somatic cells. Nat. Genet. *47*, 1402–1407.

Alexandrov, L.B., Kim, J., Haradhvala, N.J., Huang, M.N., Tian Ng, A.W., Wu, Y., Boot, A., Covington, K.R., Gordenin, D.A., Bergstrom, E.N., et al.; PCAWG Mutational Signatures Working Group; PCAWG Consortium (2020). The repertoire of mutational signatures in human cancer. Nature *578*, 94–101.

Armenia, J., Wankowicz, S.A.M., Liu, D., Gao, J., Kundra, R., Reznik, E., Chatila, W.K., Chakravarty, D., Han, G.C., Coleman, I., et al.; PCF/SU2C International Prostate Cancer Dream Team (2018). The long tail of oncogenic drivers in prostate cancer. Nat. Genet. *50*, 645–651.

Attard, G., Parker, C., Eeles, R.A., Schröder, F., Tomlins, S.A., Tannock, I., Drake, C.G., and de Bono, J.S. (2016). Prostate cancer. Lancet *387*, 70–82.

Bates, D., Mächler, M., Bolker, B., and Walker, S. (2015). Fitting Linear Mixed-Effects Models Using lme4. J. Stat. Softw. *67*, 1–48.

Behjati, S., Huch, M., van Boxtel, R., Karthaus, W., Wedge, D.C., Tamuri, A.U., Martincorena, I., Petljak, M., Alexandrov, L.B., Gundem, G., et al. (2014). Genome sequencing of normal cells reveals developmental lineages and mutational processes. Nature *513*, 422–425.

Blackwood, J.K., Williamson, S.C., Greaves, L.C., Wilson, L., Rigas, A.C., Sandher, R., Pickard, R.S., Robson, C.N., Turnbull, D.M., Taylor, R.W., and Heer, R. (2011). In situ lineage tracking of human prostatic epithelial stem cell fate reveals a common clonal origin for basal and luminal cells. J. Pathol. *225*, 181–188.

Blokzijl, F., de Ligt, J., Jager, M., Sasselli, V., Roerink, S., Sasaki, N., Huch, M., Boymans, S., Kuijk, E., Prins, P., et al. (2016). Tissue-specific mutation accumulation in human adult stem cells during life. Nature *538*, 260–264.

Boutros, P.C., Fraser, M., Harding, N.J., de Borja, R., Trudel, D., Lalonde, E., Meng, A., Hennings-Yeomans, P.H., McPherson, A., Sabelnykova, V.Y., et al. (2015). Spatial genomic heterogeneity within localized, multifocal prostate cancer. Nat. Genet. *47*, 736–745.

Boyd, L.K., Mao, X., Xue, L., Lin, D., Chaplin, T., Kudahetti, S.C., Stankiewicz, E., Yu, Y., Beltran, L., Shaw, G., et al. (2012). High-resolution genome-wide copy-number analysis suggests a monoclonal origin of multifocal prostate cancer. Genes Chromosomes Cancer *51*, 579–589.

CellPress
OPEN ACCESS

Brunner, S.F., Roberts, N.D., Wylie, L.A., Moore, L., Aitken, S.J., Davies, S.E., Sanders, M.A., Ellis, P., Alder, C., Hooks, Y., et al. (2019). Somatic mutations and clonal dynamics in healthy and cirrhotic human liver. Nature *574*, 538–542.

Cancer Genome Atlas Research Network (2015). The Molecular Taxonomy of Primary Prostate Cancer. Cell *163*, 1011–1025.

CASM/CancerIT (2019). BRASS - Breakpoints via assembly. https://github.com/cancerit/BRASS.

Collins, A.T., Habib, F.K., Maitland, N.J., and Neal, D.E. (2001). Identification and isolation of human prostate epithelial stem cells based on alpha(2) beta(1)-integrin expression. J. Cell Sci. *114*, 3865–3872.

Cooper, C.S., Eeles, R., Wedge, D.C., Van Loo, P., Gundem, G., Alexandrov, L.B., Kremeyer, B., Butler, A., Lynch, A.G., Camacho, N., et al.; ICGC Prostate Group (2015). Analysis of the genetic phylogeny of multifocal prostate cancer identifies multiple independent clonal expansions in neoplastic and morphologically normal prostate tissue. Nat. Genet. *47*, 367–372.

Cunha, G.R., Donjacour, A.A., Cooke, P.S., Mee, S., Bigsby, R.M., Higgins, S.J., and Sugimura, Y. (1987). The endocrinology and developmental biology of the prostate. Endocr. Rev. *8*, 338–362.

Cunha, G.R., Vezina, C.M., Isaacson, D., Ricke, W.A., Timms, B.G., Cao, M., Franco, O., and Baskin, L.S. (2018). Development of the human prostate. Differentiation *103*, 24–45.

Drost, J., Karthaus, W.R., Gao, D., Driehuis, E., Sawyers, C.L., Chen, Y., and Clevers, H. (2016). Organoid culture systems for prostate epithelial and cancer tissue. Nat. Protoc. *11*, 347–358.

Farmery, J.H.R., Smith, M.L., and Lynch, A.G.; NIHR BioResource - Rare Diseases (2018). Telomerecat: A ploidy-agnostic method for estimating telomere length from whole genome sequencing data. Sci. Rep. *8*, 1300.

Fiala, J.C. (2005). Reconstruct: a free editor for serial section microscopy. J. Microsc. *218*, 52–61.

Franco, I., Johansson, A., Olsson, K., Vrtačnik, P., Lundin, P., Helgadottir, H.T., Larsson, M., Revêchon, G., Bosia, C., Pagnani, A., et al. (2018). Somatic mutagenesis in satellite cells associates with human skeletal muscle aging. Nat. Commun. *9*, 800.

Gaisa, N.T., Graham, T.A., McDonald, S.A., Poulsom, R., Heidenreich, A., Jakse, G., Knuechel, R., and Wright, N.A. (2011). Clonal architecture of human prostatic epithelium in benign and malignant conditions. J. Pathol. *225*, 172–180.

Gasi Tandefelt, D., Boormans, J., Hermans, K., and Trapman, J. (2014). ETS fusion genes in prostate cancer. Endocr. Relat. Cancer *21*, R143–R152.

Gerhardt, J., Montani, M., Wild, P., Beer, M., Huber, F., Hermanns, T., Müntener, M., and Kristiansen, G. (2012). FOXA1 promotes tumor progression in prostate cancer and represents a novel hallmark of castration-resistant prostate cancer. Am. J. Pathol. *180*, 848–861.

Goldstein, A.S., Lawson, D.A., Cheng, D., Sun, W., Garraway, I.P., and Witte, O.N. (2008). Trop2 identifies a subpopulation of murine and human prostate basal cells with stem cell characteristics. Proc. Natl. Acad. Sci. USA *105*, 20882–20887.

Gonzalez-Perez, A., Perez-Llamas, C., Deu-Pons, J., Tamborero, D., Schroeder, M.P., Jene-Sanz, A., Santos, A., and Lopez-Bigas, N. (2013). IntOGen-mutations identifies cancer drivers across tumor types. Nat. Methods *10*, 1081–1082.

Hudson, D.L., O'Hare, M., Watt, F.M., and Masters, J.R. (2000). Proliferative heterogeneity in the human prostate: evidence for epithelial stem cells. Lab. Invest. *80*, 1243–1250.

ICGC/TCGA Pan-Cancer Analysis of Whole Genomes Consortium (2020). Pan-cancer analysis of whole genomes. Nature *578*, 82–93.

Karthaus, W.R., Iaquinta, P.J., Drost, J., Gracanin, A., van Boxtel, R., Wongvipat, J., Dowling, C.M., Gao, D., Begthel, H., Sachs, N., et al. (2014). Identification of multipotent luminal progenitor cells in human prostate organoid cultures. Cell *159*, 163–175.

Karthaus, W.R., Hofree, M., Choi, D., Linton, E.L., Turkekul, M., Bejnood, A., Carver, B., Gopalan, A., Abida, W., Laudone, V., et al. (2020). Regenerative potential of prostate luminal cells revealed by single-cell analysis. Science *368*, 497–505.

Kimura, M. (1969). The number of heterozygous nucleotide sites maintained in a finite population due to steady flux of mutations. Genetics *61*, 893–903.

Lee-Six, H., Øbro, N.F., Shepherd, M.S., Grossmann, S., Dawson, K., Belmonte, M., Osborne, R.J., Huntly, B.J.P., Martincorena, I., Anderson, E., et al. (2018). Population dynamics of normal human blood inferred from somatic mutations. Nature *561*, 473–478.

Lee-Six, H., Olafsson, S., Ellis, P., Osborne, R.J., Sanders, M.A., Moore, L., Georgakopoulos, N., Torrente, F., Noorani, A., Goddard, M., et al. (2019). The landscape of somatic mutation in normal colorectal epithelial cells. Nature *574*, 532–537.

Leong, K.G., Wang, B.E., Johnson, L., and Gao, W.Q. (2008). Generation of a prostate from a single adult stem cell. Nature *456*, 804–808.

Li, H. (2013). Aligning sequence reads, clone sequences and assembly contigs with BWA-MEM. arXiv, arXiv:13033997. https://arxiv.org/abs/1303.3997.

Li, H., Handsaker, B., Wysoker, A., Fennell, T., Ruan, J., Homer, N., Marth, G., Abecasis, G., and Durbin, R.; 1000 Genome Project Data Processing Subgroup (2009). The Sequence Alignment/Map format and SAMtools. Bioinformatics *25*, 2078–2079.

Lindberg, J., Klevebring, D., Liu, W., Neiman, M., Xu, J., Wiklund, P., Wiklund, F., Mills, I.G., Egevad, L., and Grönberg, H. (2013). Exome sequencing of prostate cancer supports the hypothesis of independent tumour origins. Eur. Urol. *63*, 347–353.

Martincorena, I. (2019). dndscv: Poisson-based dN/dS models to quantify natural selection in somatic evolution. https://rdrr.io/github/im3sanger/dndscv/.

Martincorena, I., Roshan, A., Gerstung, M., Ellis, P., Van Loo, P., McLaren, S., Wedge, D.C., Fullam, A., Alexandrov, L.B., Tubio, J.M., et al. (2015). Tumor evolution. High burden and pervasive positive selection of somatic mutations in normal human skin. Science *348*, 880–886.

Martincorena, I., Raine, K.M., Gerstung, M., Dawson, K.J., Haase, K., Van Loo, P., Davies, H., Stratton, M.R., and Campbell, P.J. (2017). Universal Patterns of Selection in Cancer and Somatic Tissues. Cell *171*, 1029–1041.e21.

Martincorena, I., Fowler, J.C., Wabik, A., Lawson, A.R.J., Abascal, F., Hall, M.W.J., Cagan, A., Murai, K., Mahbubani, K., Stratton, M.R., et al. (2018). Somatic mutant clones colonize the human esophagus with age. Science *362*, 911–917.

McNeal, J.E. (1968). Regional morphology and pathology of the prostate. Am. J. Clin. Pathol. *49*, 347–357.

Moad, M., Hannezo, E., Buczacki, S.J., Wilson, L., El-Sherif, A., Sims, D., Pickard, R., Wright, N.A., Williamson, S.C., Turnbull, D.M., et al. (2017). Multipotent Basal Stem Cells, Maintained in Localized Proximal Niches, Support Directed Long-Ranging Epithelial Flows in Human Prostates. Cell Rep. *20*, 1609–1622.

Moore, L., Leongamornlert, D., Coorens, T.H.H., Sanders, M.A., Ellis, P., Dawson, K., Maura, F., Nangalia, J., Tarpey, P.S., Brunner, S.F., et al. (2018). The mutational landscape of normal human endometrial epithelium. Nature *580*, 640–646.

Moore, L., Leongamornlert, D., Coorens, T.H.H., Sanders, M.A., Ellis, P., Dentro, S.C., Dawson, K.J., Butler, T., Rahbari, R., Mitchell, T.J., et al. (2020). The mutational landscape of normal human endometrial epithelium. Nature *580*, 640–646.

Nik-Zainal, S., Alexandrov, L.B., Wedge, D.C., Van Loo, P., Greenman, C.D., Raine, K., Jones, D., Hinton, J., Marshall, J., Stebbings, L.A., et al.; Breast Cancer Working Group of the International Cancer Genome Consortium (2012a). Mutational processes molding the genomes of 21 breast cancers. Cell *149*, 979–993.

Nik-Zainal, S., Van Loo, P., Wedge, D.C., Alexandrov, L.B., Greenman, C.D., Lau, K.W., Raine, K., Jones, D., Marshall, J., Ramakrishna, M., et al.; Breast Cancer Working Group of the International Cancer Genome Consortium (2012b). The life history of 21 breast cancers. Cell *149*, 994–1007.

Ousset, M., Van Keymeulen, A., Bouvencourt, G., Sharma, N., Achouri, Y., Simons, B.D., and Blanpain, C. (2012). Multipotent and unipotent progenitors contribute to prostate postnatal development. Nat. Cell Biol. *14*, 1131–1138.

Raine, K.M., Hinton, J., Butler, A.P., Teague, J.W., Davies, H., Tarpey, P., Nik-Zainal, S., and Campbell, P.J. (2015). cgpPindel: Identifying Somatically

Acquired Insertion and Deletion Events from Paired End Sequencing. Curr. Protoc. Bioinformatics *52*, 15.17.11–15.17.12.

Raine, K.M., Van Loo, P., Wedge, D.C., Jones, D., Menzies, A., Butler, A.P., Teague, J.W., Tarpey, P., Nik-Zainal, S., and Campbell, P.J. (2016). ascatNgs: Identifying Somatically Acquired Copy-Number Alterations from Whole-Genome Sequencing Data. Curr. Protoc. Bioinformatics *56*, 15.19.11–15.19.17.

R Core Team (2018). R: A Language and Environment for Statistical Computing (R Foundation for Statistical Computing).

Richardson, G.D., Robson, C.N., Lang, S.H., Neal, D.E., Maitland, N.J., and Collins, A.T. (2004). CD133, a novel marker for human prostatic epithelial stem cells. J. Cell Sci. *117*, 3539–3545.

Sievert, C. (2018). Interactive web-based data visualization with R, plotly, and shiny (CRC Press).

Suda, K., Nakaoka, H., Yoshihara, K., Ishiguro, T., Tamura, R., Mori, Y., Yamawaki, K., Adachi, S., Takahashi, T., Kase, H., et al. (2018). Clonal Expansion and Diversification of Cancer-Associated Mutations in Endometriosis and Normal Endometrium. Cell Rep. *24*, 1777–1789.

Tate, J.G., Bamford, S., Jubb, H.C., Sondka, Z., Beare, D.M., Bindal, N., Boutselakis, H., Cole, C.G., Creatore, C., Dawson, E., et al. (2019). COSMIC: the Catalogue Of Somatic Mutations In Cancer. Nucleic Acids Res. *47* (D1), D941–D947.

Toivanen, R., and Shen, M.M. (2017). Prostate organogenesis: tissue induction, hormonal regulation and cell type specification. Development *144*, 1382–1398.

Wang, Y., Hayward, S., Cao, M., Thayer, K., and Cunha, G. (2001). Cell differentiation lineage in the prostate. Differentiation *68*, 270–279.

Wang, X., Kruithof-de Julio, M., Economides, K.D., Walker, D., Yu, H., Halili, M.V., Hu, Y.P., Price, S.M., Abate-Shen, C., and Shen, M.M. (2009). A luminal epithelial stem cell that is a cell of origin for prostate cancer. Nature *461*, 495–500.

Woodworth, M.B., Girskis, K.M., and Walsh, C.A. (2017). Building a lineage from single cells: genetic techniques for cell lineage tracking. Nat. Rev. Genet. *18*, 230–244.

Xue, Y., Sonke, G., Schoots, C., Schalken, J., Verhofstad, A., de la Rosette, J., and Smedts, F. (2001). Proliferative activity and branching morphogenesis in the human prostate: a closer look at pre- and postnatal prostate growth. Prostate *49*, 132–139.

Yizhak, K., Aguet, F., Kim, J., Hess, J.M., Kübler, K., Grimsby, J., Frazer, R., Zhang, H., Haradhvala, N.J., Rosebrock, D., et al. (2019). RNA sequence analysis reveals macroscopic somatic clonal expansion across normal tissues. Science *364*, eaaw0726.

Yokoyama, A., Kakiuchi, N., Yoshizato, T., Nannya, Y., Suzuki, H., Takeuchi, Y., Shiozawa, Y., Sato, Y., Aoki, K., Kim, S.K., et al. (2019). Age-related remodelling of oesophageal epithelia by mutated cancer drivers. Nature *565*, 312–317.

Yoshida, K., Gowers, K.H.C., Lee-Six, H., Chandrasekharan, D.P., Coorens, T., Maughan, E.F., Beal, K., Menzies, A., Millar, F.R., Anderson, E., et al. (2020). Tobacco smoking and somatic mutations in human bronchial epithelium. Nature *578*, 266–272.

Zaichick, S., and Zaichick, V. (2013). Relations of morphometric parameters to zinc content in paediatric and nonhyperplastic young adult prostate glands. Andrology *1*, 139–146.

STAR★METHODS

KEY RESOURCES TABLE

REAGENT or RESOURCE	SOURCE	IDENTIFIER
Biological samples		
Snap-frozen human whole prostate tissues	Newcastle University	N/A
Snap-frozen human prostate post-mortem biopsies	Amsbio Biorepository	https://www.amsbio.com/products/biorepository
Chemicals, peptides, and recombinant proteins		
PAXgene Tissue FIX &	QIAGEN	Cat No.: 765312
PAXgene Tissue STABILIZER Concentrate	QIAGEN	Cat No.: 765512
Critical commercial assays		
Arcturus PicoPure DNA Extraction Kit	Thermo Fisher Scientific	Cat No.: KIT0103
NEBNext Ultra II FS Reaction	NEB	Cat No.: E7805L
Agilent High-Sensitivity DNA Kit	Agilent	Cat. No. 5067-4626
Deposited data		
WGS Sequencing Data	European Genome-Phenome Archive	EGAD00001006591
Oligonucleotides		
Duplexed adapters (HPLC grade, '*' represents phosphorothioate modification):<br>5′-ACACTCTTTCCCTACACGACGCTCTTCCGATC*T-3′<br>5′-phos-GATCGGAAGAGCGGTTCAGCAGGAATGCC GAG-3′	IDT	N/A
Software and algorithms		
Variant calling pipelines:<br>ASCAT (copy number)<br>BRASS (structural variation)<br>Caveman (base substitutions)<br>Pindel (indels)	Wellcome Sanger Institute github repository	https://github.com/cancerit
Samtools	Li et al., 2009	http://www.htslib.org/download/
Bwa-mem	Li, 2013	https://github.com/lh3/bwa
NDP.view2 Viewing software	Hamamatsu	https://www.hamamatsu.com/eu/en/product/type/U12388-01/index.html
Reconstruct	Fiala, 2005	https://www.bu.edu/neural/Reconstruct.html
R version 3.5.0 (critical packages as cited in text provided below)	R Core Team, 2018	https://www.r-project.org/about.html
R package plotly	Sievert, 2018	https://cran.r-project.org/web/packages/plotly/index.html
R package dndscv	Martincorena, 2019	https://github.com/im3sanger/dndscv
R package lme4	Bates et al., 2015	https://cran.r-project.org/web/packages/lme4/lme4.pdf
SigProfiler v.2.1	Alexandrov et al., 2020	https://github.com/AlexandrovLab/SigProfilerExtractor
Telomerecat	Farmery et al., 2018	https://pypi.org/project/telomerecat/
Miscellaneous code for data filtering, data input preparation, visualization, and regressions in R	This paper	https://github.com/safgrossmann/prostate-pub-repo
Other		
Data visualization websites	This paper	https://sg18.shinyapps.io/RightStructure_TreeAnd3DModel/; https://sg18.shinyapps.io/LeftStructure_TreeAnd3DModel/

RESOURCE AVAILABILITY

Lead contact

Further information and requests for resources and reagents should be directed to and will be fulfilled by the lead contact, Peter J. Campbell (pc8@sanger.ac.uk).

Materials availability

This study did not generate new unique reagents.

Data and code availability

The datasets supporting the current study have been deposited in the European Genome-Phenome Archive public repository with accession number EGAD00001006591.

All code central to this analysis is publicly available and is appropriately cited in the main manuscript or STAR Methods – Method Details. Additional code for data filtering, data input preparation, visualization, and linear regression is provided on https://github.com/safgrossmann/prostate-pub-repo.

EXPERIMENTAL MODEL AND SUBJECT DETAILS

A snap-frozen whole prostate sample from a 59-year-old donor was chosen for the 3D reconstruction and phylogenetic analysis of long-ranging glandular subunits (referred to as PD40870). This donor of European descent had no family history of prostate cancer. After presentation with visible haematuria, the investigations revealed micropapillary variant bladder cancer, stage pT1. PSA was 1.8 and a clinically benign prostate was noted. He underwent radical cystoprostatectomy and ileal conduit formation. The prostate size was 30 cm^3 and histologically, there were no BPH adenomas, nor was there prostatitis and no foci of cancer were seen.

Additional snap-frozen prostate biopsies were obtained from seven additional donors to explore the mutational landscape and to infer the relationship between mutation accumulation and aging in normal prostate epithelium (Table S1).

All human samples were obtained, stored and processed with appropriate ethical approval granted by HTA/NRES Committee North East – Newcastle & North Tyneside 1 (HTA 12534 Newcastle University/12/NE/0395) or the London-Surrey Research Ethics Committee (17/LO/1801).

METHOD DETAILS

Sample Preparation for Laser-Capture Microscopy

The frozen prostate samples were equilibrated to 0°C before fixation in PAXgene Tissue Fix and the corresponding PAXgene Tissue Stabilizer (PreAnalytiX). Subsequently, the whole prostate sample was cut longitudinally into two 10 mm blocks. The prostate biopsies were of variable thickness between 2 – 5 mm and could directly be subjected to paraffin embedding using a Sakura Tissue Tek VIP 6 Processor with standard histological tissue processing protocols (1 cycle of 90% EtOH/4h/35°C, 3 cycles of 100% EtOH/4h/35°C, 3 cycles of Xylene/4h/35°C, 4 cycles of paraffin/4h/60°C). The paraffin-embedded prostate blocks were sectioned to derive 10μm thick cross-sections of the whole prostate or biopsy region and mounted onto PEN-membrane slides (Leica). Individual sections were stained with hematoxylin and eosin (H&E) by sequential immersion into: the xylene (two minutes, twice), ethanol (100%, 1 minute, twice), ethanol (70%, 1 minute, once), deionised water (1 minute, once), Gill's hematoxylin (15 s), tap water (20 s, twice), eosin (6 s, once), tap water (15 s, once), ethanol (70%, 15 s, twice), ethanol (100%, 30 s, twice), and xylene (15 s, twice). The stained sections were dipped in the xylene-substitute NeoClear and temporarily coverslipped before slide-scans were made using a NanoZoomer S60 (Hamamatsu) up to a 20- or 40-fold magnification. No cancerous lesions were detected in the regions of interest that were subjected to Laser-Capture Microscopy (LCM) and whole genome sequencing (WGS).

Reconstruction of Prostatic Glandular Subunits

Prostatic glandular subunits were only reconstructed for the 59-year-old donor of the whole prostate. Low-resolution images at 10x magnification were extracted in jpg format from the high-resolution digital slide scans to be able to perform image registration on 671 sequential whole prostate images. Intensity-based automatic rigid image registration was performed within MATLAB. The aligned images were screened for long-ranging prostatic glandular subunits and series of several hundred image files containing the ductal trees of interest were imported into the serial section microscopy editor Reconstruct (Fiala, 2005). Branching of prostatic ductal trees was followed in proximal to distal fashion and regions of interest were annotated for subsequent LCM microdissection. Three-dimensional structures of LCM microdissections and their histological connection through the ductal network were visualized in R using the plotly package (Sievert, 2018). Two-dimensional projections were generated using metric multidimensional scaling (MDS) in R.

Laser-Capture Microscopy and Whole-Genome Sequencing

Prostatic ducts and acini of interest were dissected using an LMD7 laser-capture microscope (Leica) and collected into separate wells of a 96-well plate. Regions of interest were cut from up to three adjacent sections to yield an approximate total of 200 – 2000 cells epithelial cells per well. For the 59-year-old-donor of the whole prostate, most LCM microdissections were derived

from the reconstructed glandular subunits. Ductal and acinar regions of interest from the remaining donors were selected based on benign histological appearance and appropriate cell number for microdissection without detailed knowledge about their morphological relationship.

The DNA was extracted using the Arcturus PicoPure DNA Extraction Kit (Thermo Fisher Scientific) according to the manufacturer's instructions. Bespoke low-input WGS libraries were prepared as described earlier (Lee-Six et al., 2019). Typically, six to eight samples were multiplexed and sequenced on the same total number of Illumina HiSeq X lanes to generate 150 bp paired-end reads.

Genome Alignment and Variant Calling

The WGS data was aligned against the GRCh37 genome using bwa mem (Li, 2013). Base substitutions were called using Caveman against a donor-matched stromal prostate sample with corresponding filtering as described previously (Nik-Zainal et al., 2012a). Further post hoc filters were applied to remove variants if the supporting reads had median alignments scores (ASMD) less than 140 or more than half of them were clipped during genome alignment. Small insertions and deletions were called using cgpPindel (Raine et al., 2015). Copy numbers were called using the ASCAT algorithm, assuming an expected ploidy of 2 (Raine et al., 2016). Structural variations were called using BRASS (CASM/CancerIT, 2019).

Inference of the Relationship Between Mutation Accumulation and Aging

To infer the relationship between mutation accumulation and aging, all WGS samples derived from LCM of normal prostate epithelium that were available for this study were considered. To obtain more accurate estimates for the amount of base substitutions present in every section considered, clonality and sequencing depth of the corresponding WGS data had to be considered since polyclonality or low sequencing depth will result in low estimates of the mutational burden. Sequencing depth was computed using samtools (Li et al., 2009). The degree of polyclonality was estimated by the median variant allele fraction (VAF) of all passed base substitution calls in a sample. Since a minimum of four variant reads was required during filtering, robust estimation of polyclonality c was only possible in samples with appropriate sequencing depth d with the following relationship:

$$d \geq \frac{4}{c}$$

For example, perfectly clonal diploid samples would display a median VAF and corresponding estimate of $c = 0.5$ and thus, only required a minimum depth $d = 8$, while highly polyclonal samples with $c = 0.1$ required a sequencing depth of at least $d = 40$. Only samples that displayed sufficient sequencing depth for their corresponding clonality estimates in at least 75% of the genome, were considered in the final regression analysis. This filter excluded 127 of the 409 WGS samples, which included both available samples from PD43392. Hence, the final dataset for the age regression consisted of 282 WGS samples from seven donors.

For these samples, the sensitivity for SNV calls based on clonality and sequencing depth was computed using a generalized linear model (GLM) as previously described (Brunner et al., 2019). The GLM parameters were calculated based on LCM-derived WGS data of human liver tissue. Directly adjacent tissue sections were assumed to comprise the same variants and thus, any diverging SNV calls in directly adjacent sections were attributed to polyclonality and insufficient sequencing depth. Subsequent to sensitivity estimation s, the mutational burden as estimated from Caveman and corresponding filtering b_r was corrected to yield the mutational burden b_c:

$$b_c = \frac{b_r}{s}$$

As a justification for the use of the estimated relationship between coverage, VAF and sensitivity derived in our previous liver study, we note that there are a number of similarities between the two studies –

- Exactly the same laser-capture microdissection, library synthesis and sequencing protocols were used for the two studies;
- The same variant-calling pipelines were used;
- The range of VAFs observed in the two studies was broadly similar, with some samples showing polyclonal VAFs, and others more clonal;
- The range of sequencing depths in the liver study certainly spanned those achieved in the current prostate study (there were a few samples in the liver study that had 40-60x coverage, but this was a relatively small number and were useful to fit the upper asymptote of the sensitivity curve).

The major advantage of the liver study for estimating sensitivity is that we microdissected the same x-y region from adjacent z sections of the liver and independently sequenced these in many samples – since the clonal structure does not change over the 10-20μm between adjacent sections of liver, this gives independent assessments of the same clone and therefore accurate estimates of the relationship of sensitivity to VAF and depth. The major influences on sensitivity for calling mutations are known to be VAF and depth – using the same wet and dry laboratory protocols will then ensure that the relationship of sensitivity to VAF and depth will be equivalent between the two studies.

To account for dependent multiple samples from the same donor as well as the observed distribution of mutation count data, a generalized linear mixed-effect model was fitted to infer the effect of age a on the mutational burden b_c using the R function lmer from R package lme4 with following formula (Bates et al., 2015):

$$b_c \sim a + (1 \mid Donor)$$

We tested the effects of different levels of stringency on the mutation burden versus age regression, with cut-offs of mean depth > 20, 25 and 30. In each case, we see a strong linear increase in mutation burden with age, and this remains highly statistically significant in each case. The confidence intervals for the slope widen with increasing stringency (as expected for the smaller number of samples included in the regression), but the point estimates are always included within the 95% confidence interval quoted in the paper.

Mutational Spectra and Signature Extraction

Mutational spectra for base substitutions, corresponding signature extraction and comparison to SBS signatures from the ICGC PCAWG Platinum release was performed using SigProfiler v.2.1 (Alexandrov et al., 2020). *De novo*-extracted signatures were compared to the SBS signatures from the ICGC PCAWG Platinum release and considered significant if the combination of exposures and the *de novo*-extracted signatures displayed cosine similarity values greater than 0.95.

Detection of Positive Selection and Driver Mutations

Positive selection of mutations was evaluated using a dN/dS approach within the R package dndscv (Martincorena, 2019). This method compares the amount of non-synonymous to synonymous mutations within coding regions and accounts for local mutation rates as well as for the globally observed trinucleotide context of mutations. An excess of non-synonymous compared to synonymous mutations relative to what is expected by chance indicates positive selection, while a corresponding depletion would suggest negative selection (Martincorena et al., 2017).

In addition to the unbiased detection by the dN/dS approach, genic base substitutions resulting in missense, nonsense or essential splice site mutations were intersected with the most comprehensive catalog of base substitution driver mutations in prostate cancer comprising 97 significantly mutated genes (SMGs) as published by Armenia and colleagues in 2018 (Armenia et al., 2018). The observed change was annotated as a known driver mutation if it was present within the discovery set of Armenia and colleagues or within hotspots as annotated by the COSMIC Cancer Gene Census or the IntOGen database (Armenia et al., 2018; Gonzalez-Perez et al., 2013; Tate et al., 2019).

Telomere Length Estimation

Telomere lengths were estimated from genomic alignments using the Telomerecat bam2length command with 100 iterations for estimating the impact of insert size distribution and default parameters otherwise (Farmery et al., 2018).

Lineage Tree Inference

Base substitution profiles from LCM microdissections of reconstructed ductal subunits were used to infer their phylogenetic relationships. Adhering to the principle of the infinite site model of molecular evolution, it was assumed that only one independent mutation occurred per locus and was subsequently maintained in all cellular progeny (Kimura, 1969). For perfectly clonal samples, binary mutation calls can directly be used to infer the corresponding lineage tree. However, most microdissection samples were found to be oligo- or polyclonal. Therefore, not all mutations called in one microdissection derive from the same most recent common ancestor (MRCA).

The VAF distribution of base substitutions within one sample provides information about the clonal origin of these mutations. Discrete clusters of mutations observed at similar VAFs suggest a common clonal ancestor for all of these mutations. To use VAF instead of binary mutation calls for the lineage tree inference, the total read depth and the alternative allele count were obtained for all loci that were called by Caveman in at least one sample from the same prostatic ductal subunit. Formal inference of discrete clusters of mutations based on their VAFs was performed with an n-dimensional hierarchical Dirichlet process (n-HDP). The stick-breaking process of the n-HDP results in highly concentrated clusters of mutations that display similar VAF across samples as previously described (Brunner et al., 2019; Nik-Zainal et al., 2012a). Every cluster derived by the n-HDP represents an ancestral cell that was the MRCA of all cells carrying these mutations. Under the assumption that most normal epithelial cells are diploid, the median VAF *m* from all mutations associated with one n-HDP-derived cluster *hdp* directly translates into the cellular contribution *cc* per microdissection *i*:

$$cc_{hdp,i} = m_{hdp,i} * 2$$

For example, if the median VAF for one n-HDP-derived cluster corresponds to 0.5 in one microdissection, 100% of the cells in this microdissection share the corresponding ancestral cell as their MRCA.

For the phylogenetic inference, only clusters with at least 10 mutations were considered. Furthermore, up to 20 mutations from each cluster were manually inspected to identify clusters that comprised common sequencing and alignment artifacts. Using the relationship between median VAF and cellular contribution per microdissection, lineage relationships can be inferred based on the pigeonhole principle. Since the sum of cellular contributions cannot exceed 100% per microdissection, clones providing smaller cellular contributions must be subclones to n-HDP-derived clusters with greater cellular contributions. Absolute certainty for lineage nesting was assumed if two n-HDP-derived clusters exceeded 95% cellular contribution in at least one microdissection. Furthermore, lineage nesting was strongly suggested if two n-HDP-derived clusters displayed the same pattern of relative cellular contribution in all microdissections and their sum of cellular contribution was at least 75% in at least one microdissection.

QUANTIFICATION AND STATISTICAL ANALYSES

Processing of genomic data including pre-processing, alignment and variant calling was performed within the Wellcome Trust Sanger Institute Cancer IT pipelines. Software is publicly available at https://github.com/cancerit. The specific algorithms for individual steps are detailed in Method details and appropriately cited.

Mutational spectra for base substitutions, corresponding signature extraction and comparison to SBS signatures from the ICGC PCAWG Platinum release was performed using SigProfiler v.2.1 (Alexandrov et al., 2020) as specified in Method details.

All remaining statistical analyses were performed in R version 3.5.0 using core distribution functions unless otherwise indicated in the Method details (R Core Team, 2018). Statistical significance was assumed for p values < 0.05 (after correction for multiple testing if appropriate; specific details are provided in Method details).

ADDITIONAL RESOURCES

Two websites were created to explore the phylogenetic tree and a 3D map of the two main ductal structures discussed in the manuscript. Distribution and contribution of individual clones to the sampled microdissections can be visualized. Microdissections are displayed as spheres (with color and intensity according to the contribution of the selected clone) and ductal connections between the microdissections are indicated by black lines.

Right Structure: https://sg18.shinyapps.io/RightStructure_TreeAnd3DModel/
Left Structure: https://sg18.shinyapps.io/LeftStructure_TreeAnd3DModel/

Cell Stem Cell, Volume *28*

Supplemental Information

Development, maturation, and maintenance of human prostate inferred from somatic mutations

Sebastian Grossmann, Yvette Hooks, Laura Wilson, Luiza Moore, Laura O'Neill, Iñigo Martincorena, Thierry Voet, Michael R. Stratton, Rakesh Heer, and Peter J. Campbell

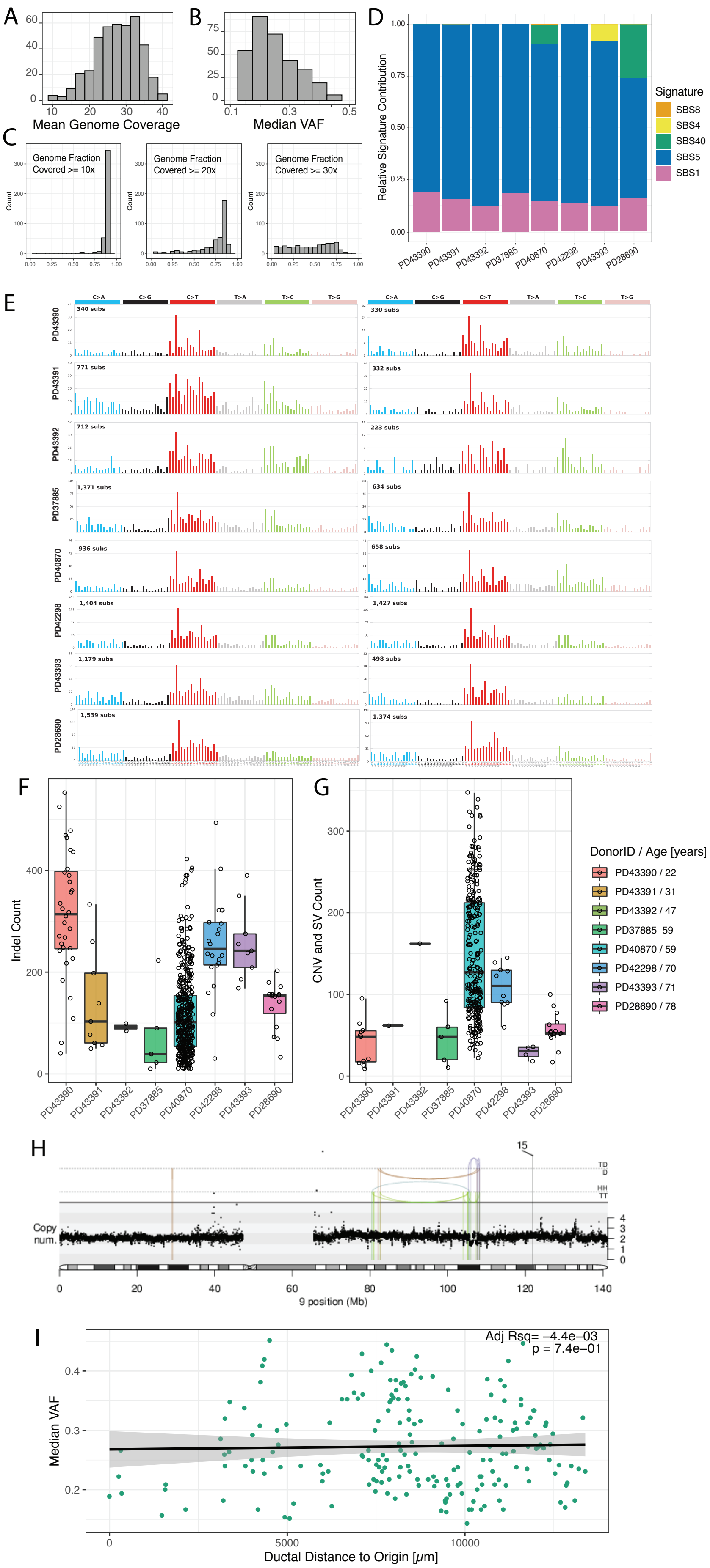
A
Mean Genome Coverage
B
Median VAF
C
Genome Fraction Covered >= 10x
Genome Fraction Covered >= 20x
Genome Fraction Covered >= 30x
Count
D
Relative Signature Contribution
Signature
SBS8
SBS4
SBS40
SBS5
SBS1
PD43390
PD43391
PD43392
PD37885
PD40870
PD42298
PD43393
PD28690
E
C>A
C>G
C>T
T>A
T>C
T>G
340 subs
330 subs
771 subs
332 subs
712 subs
223 subs
1,371 subs
634 subs
936 subs
658 subs
1,404 subs
1,427 subs
1,179 subs
498 subs
1,539 subs
1,374 subs
F
Indel Count
G
CNV and SV Count
DonorID / Age [years]
PD43390 / 22
PD43391 / 31
PD43392 / 47
PD37885 59
PD40870 / 59
PD42298 / 70
PD43393 / 71
PD28690 / 78
H
Copy num.
9 position (Mb)
I
Adj Rsq= −4.4e−03
p = 7.4e−01
Median VAF
Ductal Distance to Origin [μm]

Supplementary Figure 1 (related to Figure 1): Genome Sequencing Quality Metrics and Mutational Landscape of Normal Prostate Epithelium. (A) Mean genome coverage per sample. **(B)** Median variant allele fraction (VAF) of single base substitutions as a measure of clonality per microdissection. **(C)** Genome Fraction per sample with coverage of at least 10x/20x/30x. **(D)** Relative contribution of deconvoluted mutational signatures per donor. The clock-like mutational signatures SBS1 and SBS5 dominate the signature landscape across all donors. **(E)** Exemplary spectral signatures of base substitutions per donor. **(F)** Count of small insertions and deletions per sample. **(G)** Only few, short copy number variants and very rare structural variants were detected in normal prostatic epithelium. **(H)** One histologically normal microdissection of prostatic epithelium contained a case of chromothripsis on chromosome 9-q. **(I)** Median VAF of base substitutions per microdissection is not correlated to the position within the ductal network.

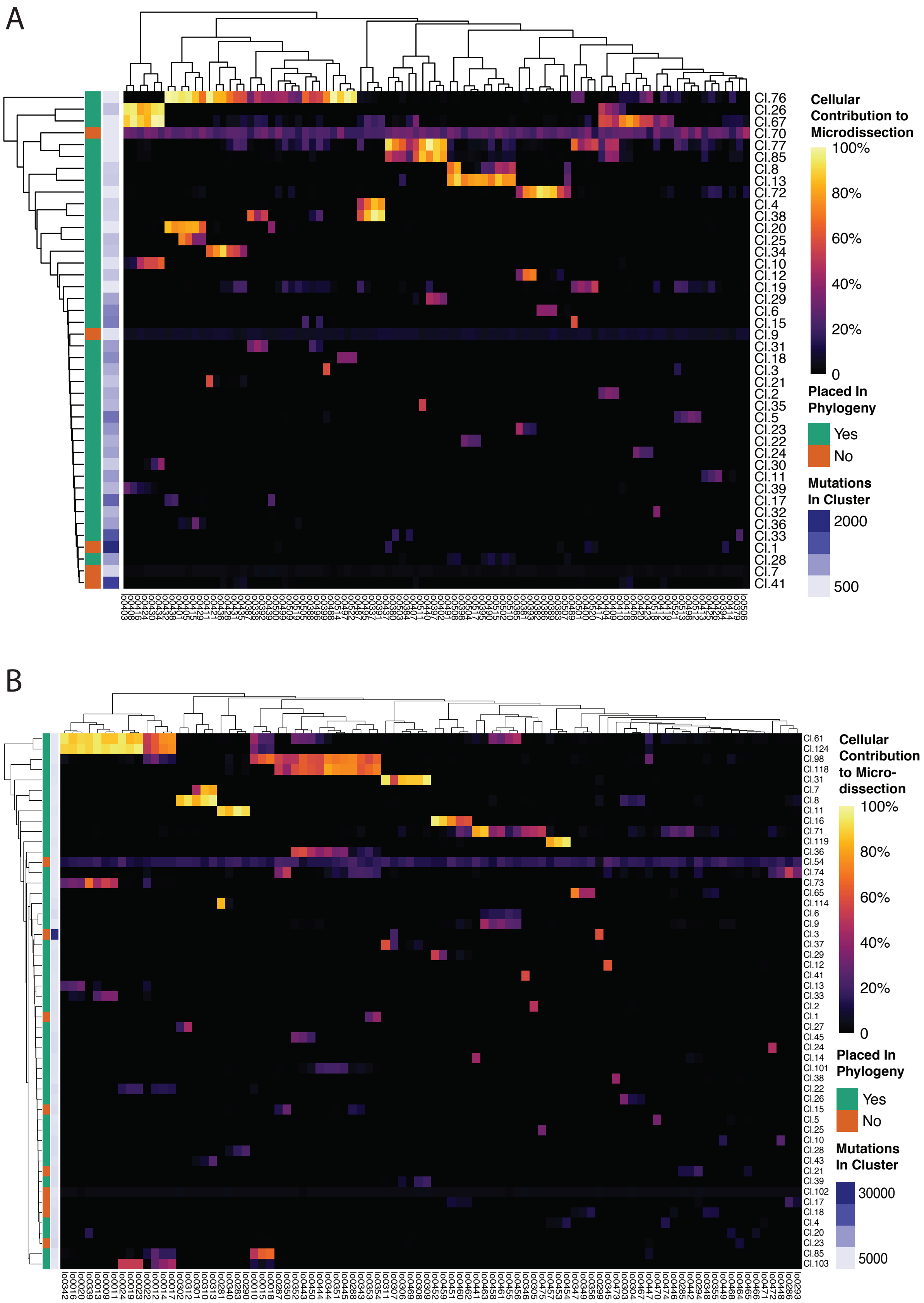
A
Cl.76
Cl.26
Cl.67
Cl.70
Cl.77
Cl.85
Cl.8
Cl.13
Cl.72
Cl.4
Cl.38
Cl.20
Cl.25
Cl.34
Cl.10
Cl.12
Cl.19
Cl.29
Cl.6
Cl.15
Cl.9
Cl.31
Cl.18
Cl.3
Cl.21
Cl.2
Cl.35
Cl.5
Cl.23
Cl.22
Cl.24
Cl.30
Cl.11
Cl.39
Cl.17
Cl.32
Cl.36
Cl.33
Cl.1
Cl.28
Cl.7
Cl.41
Cellular Contribution to Microdissection
100%
80%
60%
40%
20%
0
Placed In Phylogeny
Yes
No
Mutations In Cluster
2000
500
B
Cl.61
Cl.124
Cl.98
Cl.118
Cl.31
Cl.7
Cl.8
Cl.11
Cl.16
Cl.71
Cl.119
Cl.36
Cl.54
Cl.74
Cl.73
Cl.65
Cl.114
Cl.6
Cl.9
Cl.3
Cl.37
Cl.29
Cl.12
Cl.41
Cl.13
Cl.33
Cl.2
Cl.1
Cl.27
Cl.45
Cl.24
Cl.14
Cl.101
Cl.38
Cl.22
Cl.26
Cl.15
Cl.5
Cl.25
Cl.10
Cl.28
Cl.43
Cl.21
Cl.39
Cl.102
Cl.17
Cl.18
Cl.4
Cl.20
Cl.23
Cl.85
Cl.103
Cellular Contribution to Micro-dissection
100%
80%
60%
40%
20%
0
Placed In Phylogeny
Yes
No
Mutations In Cluster
30000
5000

Supplementary Figure 2 (related to Figure 2): Discrete Clusters of Mutations Identify Ancestral Clones that Contribute to Current Prostatic Epithelium. Clusters of mutations are identified based on VAF using an n-dimensional Hierarchical Dirichlet Process (n-HDP) clustering algorithm and constitute the rows of the heatmap. These clusters of mutations represent ancestral clones which contribute to current cells sampled in individual microdissections, which are shown in the columns of the heatmap. The median VAF of cells associated with an ancestral cluster is used to estimate the cellular contribution to each microdissection and is visualized using color intensities. The co-occurrence of multiple ancestral clones within the same microdissection sample and their corresponding cellular contribution was used to construct the lineage tree for individual glandular subunits. **(A) Heatmap for the left-hand side structure.** Cluster 70, 9 and 7 include nearly exclusively artefactual mutations and were excluded from the lineage tree. Furthermore, cluster 1 and 41 are insufficiently split by the clustering algorithm used and could not be placed unambiguously. **(B) Heatmap for the right-hand side structure.** Cluster 54 and 102 contain artefactual mutations. Seven additional clusters could not be placed onto the lineage tree unambiguously.

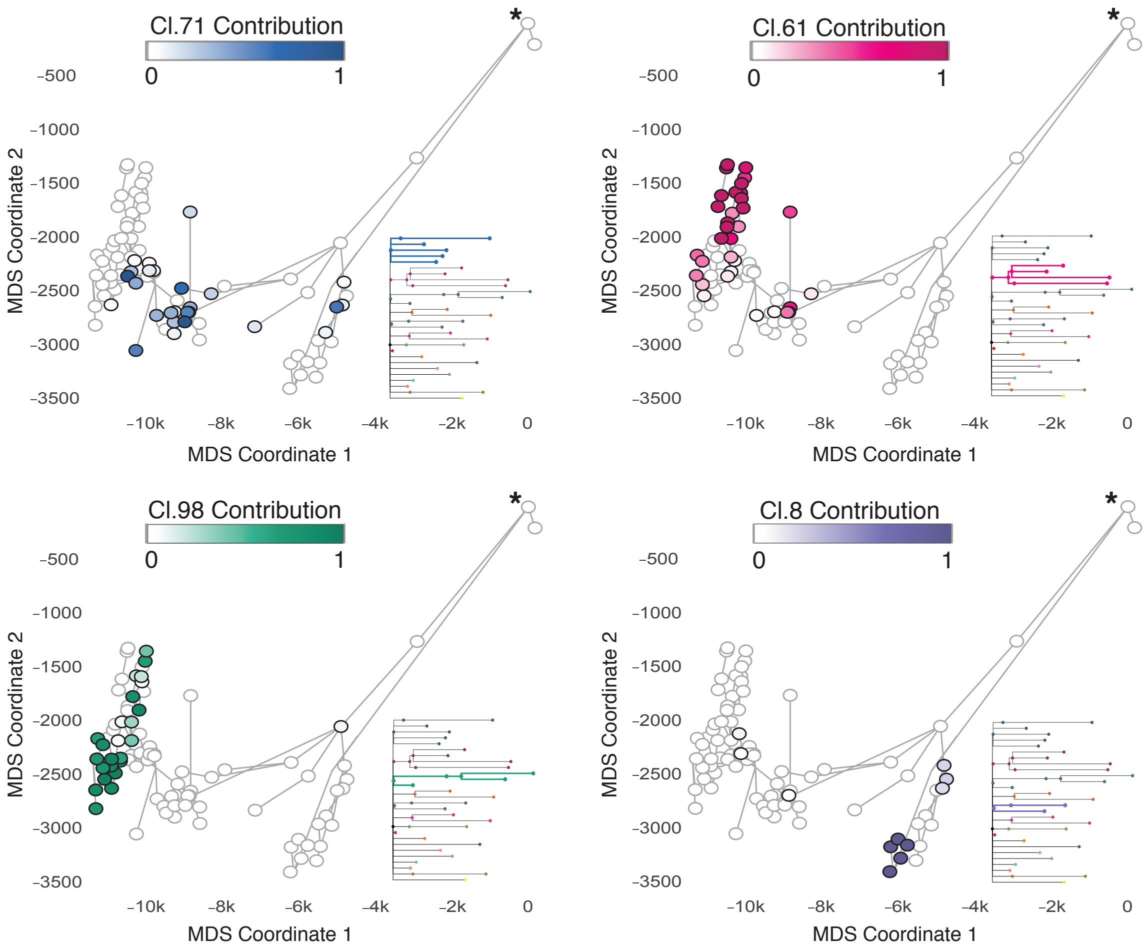

Supplementary Figure 3 (related to Figure 3): Cellular Contribution of Embryonic Clones for Glandular Subunit on the Right-Hand Side. The cellular contribution of four ancestral clones from embryonic development is displayed for the glandular subunit from the right-hand side. Each circle marks a microdissection and the ductal connection is indicated by grey lines. Microdissections with positive contribution from the clone are circled in black, while those with zero contribution are circled in grey. The most urethra-proximal microdissection is marked with an asterisk. Three embryonic clones display a wide and overlapping spatial distribution. The clone represented by cluster 8 is confined to a single sampled branch of the glandular network.

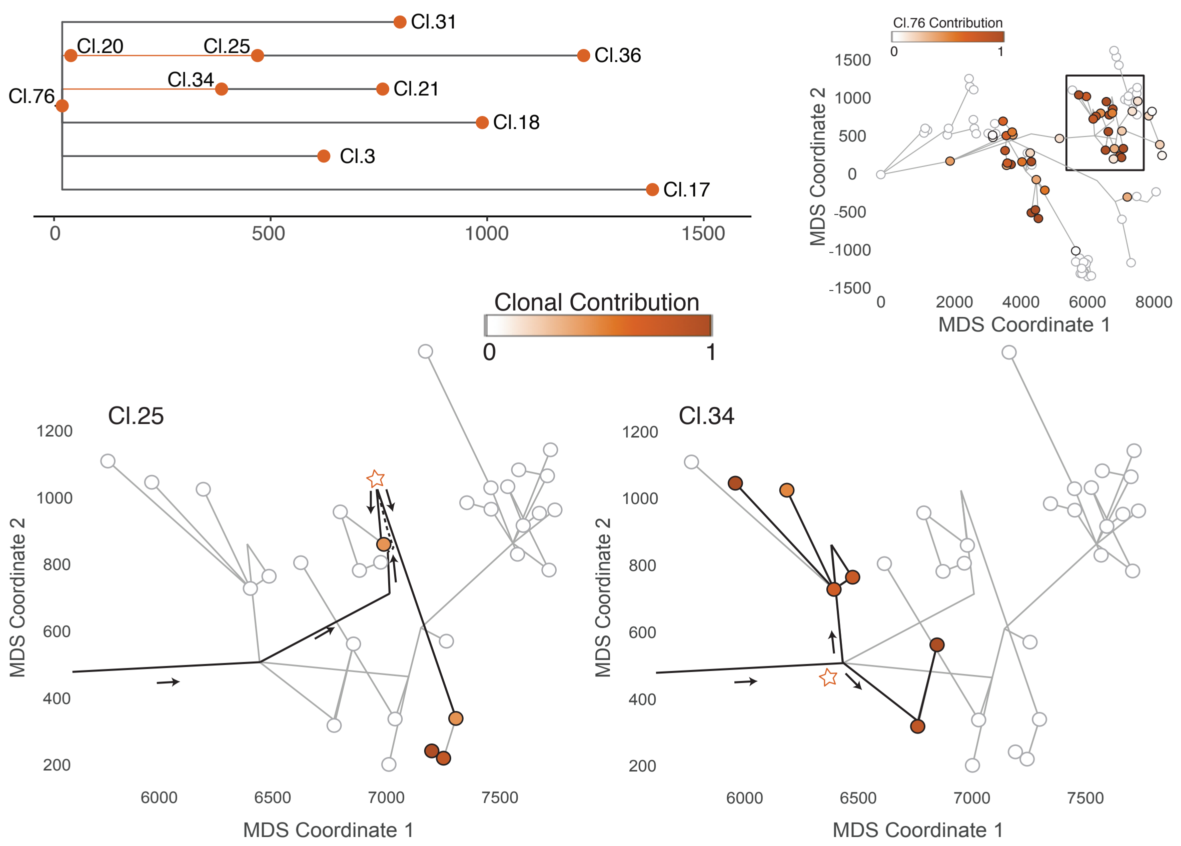

Supplementary Figure 4 (related to Figure 4): Cellular Contribution of Pubertal Clones Within the Ductal Subunit on the Left-Hand Side. The cellular contribution of pubertal clones represented by cluster 25 and 34 within the clonal territory of the embryonic clone represented by cluster 76 is highlighted. The proximal-to-peripheral direction of the ductal network is indicated by arrows. Both pubertal clones contribute to two distinct side-branches and their corresponding point from the main duct is highlighted with a star. Notably, in both cases the two branches seeded by the corresponding pubertal clones extend from the main duct directly on opposing sides and cannot be detected anywhere else. This strong spatial confinement suggests a localized progenitor cell before their respective pubertal expansions.

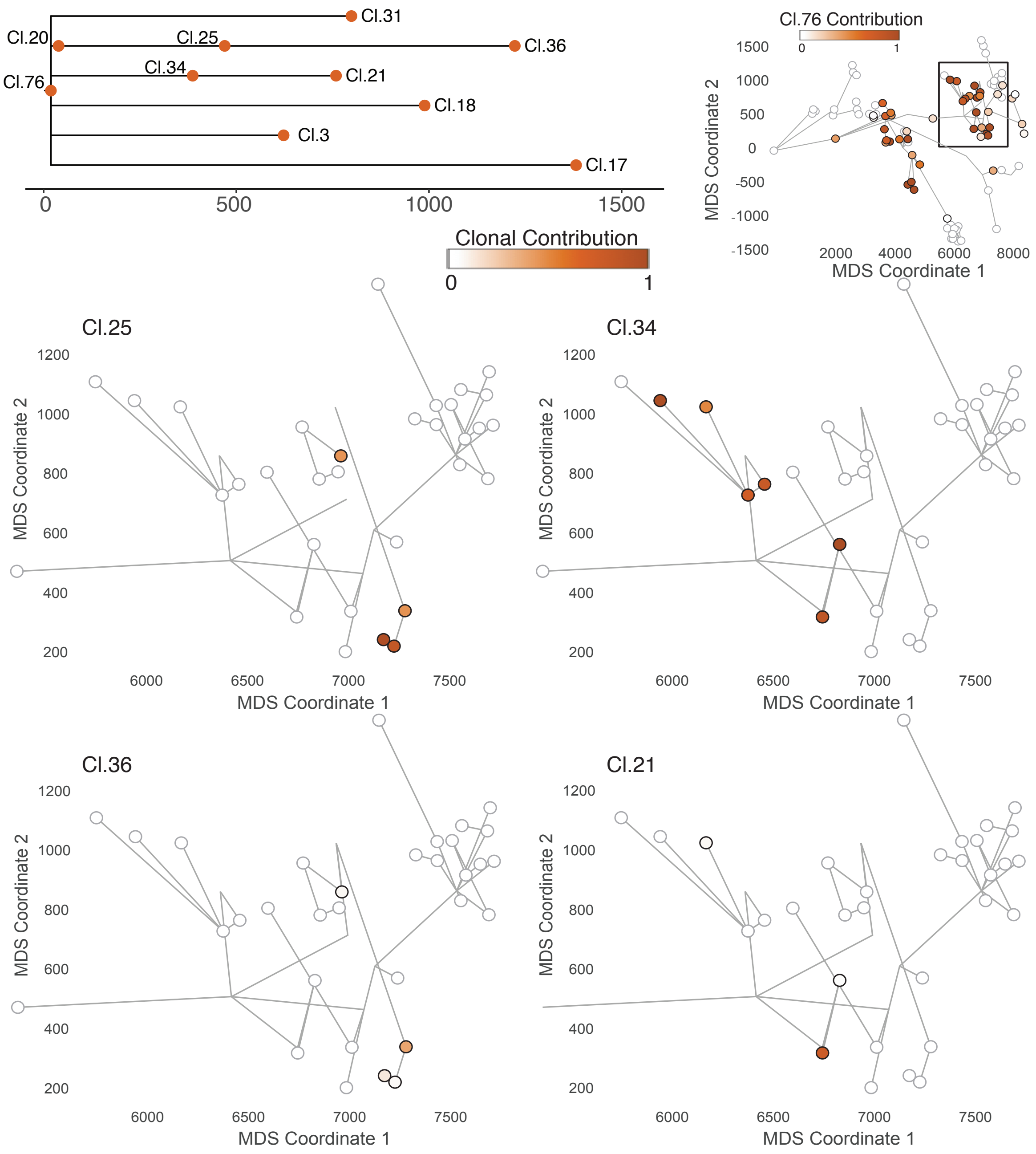

Cl.31
Cl.20
Cl.25
Cl.36
Cl.34
Cl.21
Cl.76
Cl.18
Cl.3
Cl.17
0
500
1000
1500
Cl.76 Contribution
Clonal Contribution
MDS Coordinate 1
MDS Coordinate 2
Cl.25
Cl.34
Cl.36
Cl.21

Supplementary Figure 5 (related to Figure 4): Embryonic, Pubertal and Adult Clones Occupy Increasingly Confined Territories. An example for embryonic, pubertal and adult clones within one clade of the left-hand side glandular subunit. The embryonic clone represented by cluster 76 occupies a wide spatial territory throughout most of the sampled branches. Pubertal clones represented by cluster 25 and 34 still display a confined spatial expansion with the embryonic territory. The adult clones represented by cluster 36 and 21 are only found within one or few microdissections overlapping their corresponding embryonic and pubertal clone. Notably, while most adult clones are found in peripheral microdissections, the highest cellular contribution of an adult clone is not necessarily associated with the most peripherally sampled microdissection as seen for both cluster 36 and cluster 21.

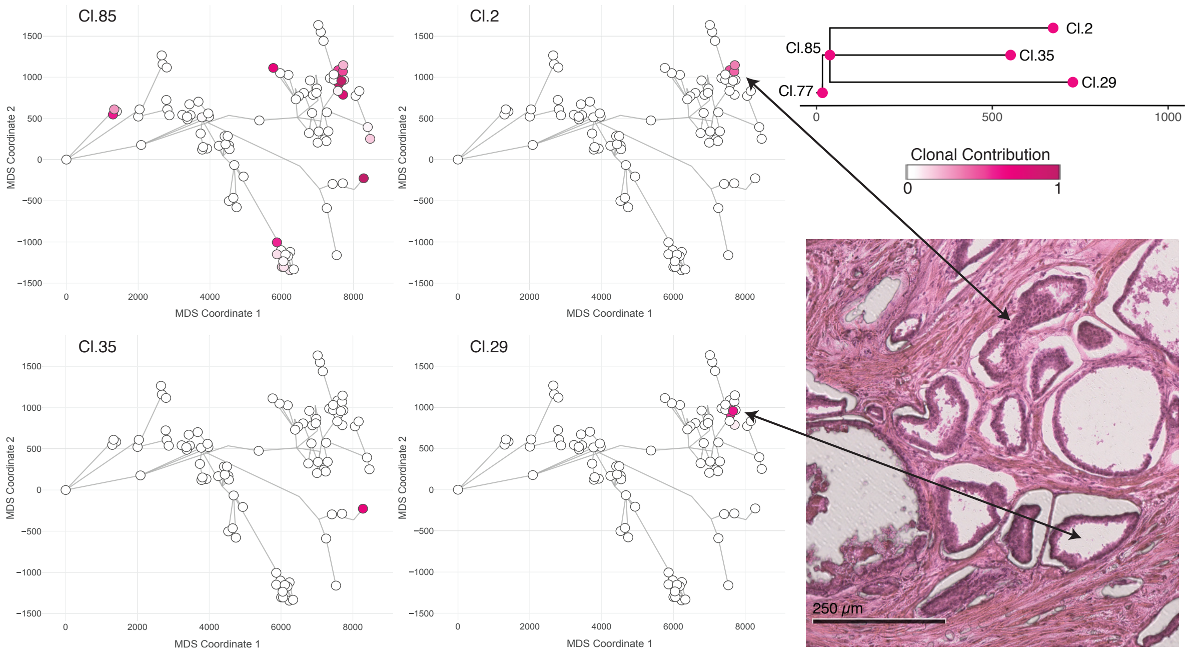

Supplementary Figure 6 (related to Figure 5): Phylogenetic Relatedness can be Independent of Spatial Proximity. An example for the glandular subunit of the left-hand side is shown. The three clones represented by cluster 2, 35 and 29 share a most recent common ancestor in the embryonic clone represented by cluster 85. Cluster 85 shows great spatial distribution across most sampled branches of the glandular subunit. While the clonal territories occupied by cluster 2 and 29 are spatially close as indicated by the structural overview as well as the histology image, the clonal territory occupied by cluster 35 is completely distinct. This example highlights the strongly localized nature of adult tissue maintenance that causes phylogenetic relationship to be largely independent of spatial proximity after embryonic and pubertal morphogenesis.

Supplementary tables

Table S1 (related to Figure 1): Overview of Available Prostate and WGS Samples. WGS from microdissections of histologically normal prostatic epithelium was obtained from eight different donors.

Donor ID	Sex	Donor Age [Years]	Sample Type	WGS Samples
PD43390	M	22	Post-mortem biopsy	32
PD43391	M	31	Post-mortem biopsy	9
PD43392	M	47	Post-mortem biopsy	2
PD37885	M	59	Post-mortem biopsy	5
PD40870	M	59	Whole prostate	319
PD42298	M	70	Biopsy	21
PD43393	M	71	Post-mortem biopsy	9
PD28690	M	78	Post-mortem biopsy	12

Table S2 (related to Figure 6): Overview of Non-synonymous Mutations in Significantly Mutated Genes in Prostate Cancer. Sixteen mutations called in microdissections from normal prostatic epithelium were found in previously published SMGs in prostate cancer. Most mutations were only called in a single microdissection.

Donor ID	Gene	Change	Impact	# of calls
PD42298	*KMT2A*	C1479W	Missense	1
PD42298	*ZNF292*	Y2193C	Missense	1
PD40870	*RPRD2*	D327G	Missense	1
PD40870	*RAG1*	R34W	Missense	1
PD40870	*RAG1*	V529M	Missense	1
PD40870	*MRE11A*	I647L	Missense	1
PD40870	*BRCA2*	F1336L	Missense	1
PD40870	*FOXA1*	R219S	Missense	5
PD40870	*NCOR1*	P1665T	Missense	1
PD40870	*NOX3*	V72I	Missense	1
PD40870	*CHD7*	R1155C	Missense	1
PD40870	*COL15A1*	A542D	Missense	2
PD40870	*AR*	.	Essential Splice	1
PD40870	*AR*	R832Q	Missense	1
PD40870	*SMARCA1*	W426*	Nonsense	1
PD28690	*PTPRC*	E362K	Missense	2
